# Supplementary material for: Insights into azalomycin F assembly-line contribute to evolution-guided polyketide synthase engineering and identification of intermodular recognition
Source: Nat Commun. 2023 Feb 4;14:612. doi: 10.1038/s41467-023-36213-9 (PMC9899208; doi:10.1038/s41467-023-36213-9)
Supplement: Supplementary file 1 — Supplementary Information [file 41467_2023_36213_MOESM1_ESM.pdf]

Supplementary Information

Insights into azalomycin F assembly-line contribute to evolution-guided polyketide synthase engineering and identification of intermodular recognition

Guifa Zhai[1,2,#], Yan Zhu[1,2,#], Guo Sun[1,2], Fan Zhou[1,2], Yangning Sun[1,2], Zhou Hong[1,2], Chuan Dong[1,2], Peter F. Leadlay[4], Kui Hong[1,2], Zixin Deng[1,2], Fuling Zhou[1], Yuhui Sun[1,2,3,*]

[1] *Department of Hematology, Zhongnan Hospital of Wuhan University, School of Pharmaceutical Sciences, Wuhan University, Wuhan 430071, People's Republic of China*

[2] *Key Laboratory of Combinatorial Biosynthesis and Drug Discovery (Ministry of Education), Wuhan University, Wuhan 430071, People's Republic of China*

[3] *Wuhan Research Center for Infectious Diseases and Cancer, Chinese Academy of Medical Sciences, Wuhan 430071, People's Republic of China*

[4] *Department of Biochemistry, University of Cambridge, Cambridge, CB2 1GA United Kingdom*

[#] *These authors contributed equally*

* *E-mail: yhsun@whu.edu.cn*

Table of Contents

I. Supplementary Tables

Supplementary Table 1. ^{1}H (600 MHz) and ^{13}C (150 MHz) NMR spectroscopic data (CH_3OH-d_4) of compound **1**.

Supplementary Table 2. ^{1}H (600 MHz) and ^{13}C (150 MHz) NMR spectroscopic data (CH_3OH-d_4) of compound **2**.

Supplementary Table 3. ^{1}H (600 MHz) and ^{13}C (150 MHz) NMR spectroscopic data (CH_3OH-d_4) of compound **3**.

Supplementary Table 4. ^{1}H (600 MHz) and ^{13}C (150 MHz) NMR spectroscopic data (CH_3OH-d_4) of compound **4**.

Supplementary Table 5. Bacterial strains used in this study.

Supplementary Table 6. Plasmids used in this study.

Supplementary Table 7. Primers used in this study.

II. Supplementary Figures

Supplementary Fig. 1. $ER_{1/2}$ domain replacements based on wild-type strain.

Supplementary Fig. 2. Comparison of structure and non-colinear enoylreduction between AZL F3a and polaramycin A PKS.

Supplementary Fig. 3. LC-ESI-HRMS analysis of polaramycin A (a) and its unsaturated analog (b) produced by *S. hygroscopicus* LP-93.

Supplementary Fig. 4. Sequence alignments of ATp-DH-KR tridomain from module 3, 5, 6, 19 and 20 that contain 5 domains.

Supplementary Fig. 5. Domain phylogenetic analysis of AZL PKS.

Supplementary Fig. 6. Domain replacements based on ΔazlB mutant.

Supplementary Fig. 7. $ER_{1/2}$-$KR_{1/2}$ didomain replacement with ER_{15}-KR_{15} based on wild-type strain.

Supplementary Fig. 8. The proposed domain topologies of module 1/2 and module 3 in the state of cross-module enoylreduction.

Supplementary Fig. 9. The structural alignment of KR domains.

III. Supplementary Notes

Supplementary Note 1. NMR spectra of compound **1**.

Supplementary Fig. 10. ^{1}H NMR spectrum (600 MHz, CD_3OD) of compound **1**.

Supplementary Fig. 11. ^{13}C NMR spectrum (150 MHz, CD_3OD) of compound **1**.

Supplementary Fig. 12. HSQC spectrum (600 MHz, CD_3OD) of compound **1**.

Supplementary Fig. 13. ^1H-^1H COSY spectrum (600 MHz, CD_3OD) of compound **1**.
Supplementary Fig. 14. HMBC spectrum (600 MHz, CD_3OD) of compound **1**.
Supplementary Fig. 15. ROESY spectrum (600 MHz, CD_3OD) of compound **1**.
Supplementary Note 2. NMR spectra of compound **2**.
Supplementary Fig. 16. ^1H NMR spectrum (600 MHz, CD_3OD) of compound **2**.
Supplementary Fig. 17. ^{13}C NMR spectrum (150 MHz, CD_3OD) of compound **2**.
Supplementary Fig. 18. HSQC spectrum (600 MHz, CD_3OD) of compound **2**.
Supplementary Fig. 19. ^1H-^1H COSY spectrum (600 MHz, CD_3OD) of compound **2**.
Supplementary Fig. 20. HMBC spectrum (600 MHz, CD_3OD) of compound **2**.
Supplementary Fig. 21. ROESY spectrum (600 MHz, CD_3OD) of compound **2**.
Supplementary Note 3. NMR spectra of compound **3**.
Supplementary Fig. 22. ^1H NMR spectrum (600 MHz, CD_3OD) of compound **3**.
Supplementary Fig. 23. ^{13}C NMR spectrum (150 MHz, CD_3OD) of compound **3**.
Supplementary Fig. 24. HSQC spectrum (600 MHz, CD_3OD) of compound **3**.
Supplementary Fig. 25. ^1H-^1H COSY spectrum (600 MHz, CD_3OD) of compound **3**.
Supplementary Fig. 26. HMBC spectrum (600 MHz, CD_3OD) of compound **3**.
Supplementary Fig. 27. ROESY spectrum (600 MHz, CD_3OD) of compound **3**.
Supplementary Note 4. NMR spectra of compound **4**.
Supplementary Fig. 28. ^1H NMR spectrum (600 MHz, CD_3OD) of compound **4**.
Supplementary Fig. 29. ^{13}C NMR spectrum (150 MHz, CD_3OD) of compound **4**.
Supplementary Fig. 30. HSQC spectrum (600 MHz, CD_3OD) of compound **4**.

Supplementary Fig. 31. ^1H-^1H COSY spectrum (600 MHz, CD_3OD) of compound **4**.

Supplementary Fig. 32. HMBC spectrum (600 MHz, CD_3OD) of compound **4**.

Supplementary Fig. 33. ROESY spectrum (600 MHz, CD_3OD) of compound **3**.

IV. Supplementary References

Supplementary Table 1. ^{1}H (600 MHz) and ^{13}C (150 MHz) NMR spectroscopic data (CH_3OH-d_4) of compound **1**.

	δ_C	δ_H		δ_C	δ_H
1	169.3, s	—	29	74.2, d	4.20 (dd, J = 9.2, 2.8 Hz, 1H)
2	126.7, s	—	30	140.2, s	—
3	140.2, d	7.12 (dd, J = 11.3, 1.1 Hz, 1H)	31	125.2, d	6.01 (dd, J = 10.8, 1.0 Hz, 1H)
4	127.6, d	6.43 (ddd, J = 15.0, 11.3, 0.6 Hz, 1H)	32	128.5, d	6.29 (ddd, J = 14.5, 10.9, 0.7 Hz, 1H)
5	146.0, d	6.06 (dd, J = 15.0, 8.7 Hz, 1H)	33	135.9, d	5.48 (dd, J = 15.0, 8.2 Hz, 1H)
6	44.4, d	2.44 (dq, J = 13.9, 6.9 Hz, 1H)	34	40.8, d	2.61 (dq, J = 14.6, 7.1 Hz, 1H)
7	75.7, d	3.77 (dd, J = 8.7, 4.1 Hz, 1H)	35	84.3, d	4.97 (d, J = 8.0 Hz, 1H)
8	39.4, t	1.76 (m, 1H), 1.48 (m, 1H)	36	133.2, s	—
9	74.8, d	3.75 (dd, J = 9.7, 2.5 Hz, 1H)	37	130.9, d	5.43 (t, J = 7.3 Hz, 1H)
10	44.6, d	1.72 (m, 1H)	38	28.3, t	2.07 (p, J = 6.8 Hz, 2H)
11	72.3, d	3.90 (m, 1H)	39	30.3, t	1.39 (m, 2H)
12	33.6, t	1.61 (m, 1H), 1.34 (m, 1H)	40	30.2, t	1.34 (m, 2H)
13	30.5, t	1.29 (m, 2H)	41	30.3, t	1.34 (m, 2H)
14	40.7, d	1.59 (m, 1H)	42	27.7, t	1.36 (m, 2H)
15	72.3, d	3.84 (m, 1H)	43	29.9, t	1,58 (m, 2H)
16	41.7, t	1.81 (m, 1H), 1.74 (m, 1H)	44	42.5, t	3.15 (t, J = 7.2 Hz, 2H)
17	99.8, s	—	45	12.8, q	1.90 (d, J = 1.0 Hz, 3H)
18	77.1, d	3.35 (d, J = 9.2 Hz, 1H)	46	17.1, q	1.11 (d, J = 6.9 Hz, 3H)
19	69.7, d	3.86 (m, 1H)	47	10.5, q	0.87 (d, J = 7.0 Hz, 3H)
20	41.2, t	1.91 (m, 1H), 1.29 (m, 1H)	48	14.9, q	0.91 (d, J = 6.8 Hz, 3H)
21	65.5, d	3.89 (m, 1H)	49	13.3, q	1.69 (d, J = 0.9 Hz, 3H)
22	41.7, t	1.77 (m, 1H), 1.69 (m, 1H)	50	17.6, q	0.97 (d, J = 6.8 Hz, 3H)
23	71.0, d	5.21 (tt, J = 8.9, 4.5 Hz, 1H)	51	12.3, q	1.66 (s, 3H)
24	44.3, t	1.58 (m, 2H)	52	158.6, s	—
25	65.7, d	4.07 (m, 1H)	53	171.5, s	—
26	46.4, t	1.49 (m, 2H)	54	45.6, t	3.21 (s, 2H)
27	66.2, d	4.05 (m, 1H)	55	174.2, s	—
28	44.4, t	1.71 (m, 2H)			

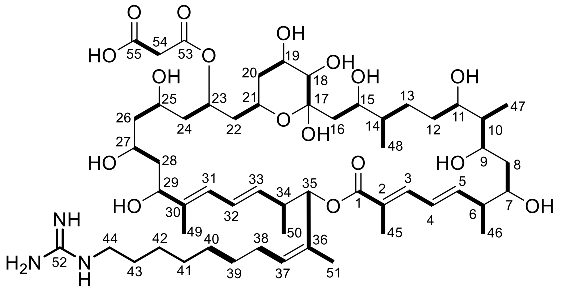

Supplementary Table 2. ^{1}H (600 MHz) and ^{13}C (150 MHz) NMR spectroscopic data (CH_3OH-d_4) of compound **2**.

	δ_C	δ_H		δ_C	δ_H
1	170.0, s	–	29	74.1, d	4.17 (d, J = 9.3 Hz, 1H)
2	126.7, s	–	30	140.0, s	–
3	140.2, d	7.08 (d, J = 11.1 Hz, 1H)	31	125.2, d	5.98 (d, J = 10.6 Hz, 1H)
4	127.6, d	6.42 (dd, J = 14.6, 11.8 Hz, 1H)	32	128.5, d	6.20 (dd, J = 14.8, 10.9 Hz, 1H)
5	146.1, d	6.04 (dd, J = 14.8, 8.9 Hz, 1H)	33	136.2, d	5.41 (dd, J = 14.9, 8.9 Hz, 1H)
6	44.5, d	2.43 (m, 1H)	34	41.0, d	2.55 (m, 1H)
7	75.8, d	3.75 (m, 1H)	35	80.6, d	4.78 (dd, J = 7.5, 3.5 Hz, 1H)
8	39.1, t	1.78 (m, 1H); 1.48 (m, 1H)	36	35.2, d	1.81 (m, 1H)
9	74.9, d	3.77 (m, 1H)	37	35.0, t	1.33 (m, 1H); 1.16 (m, 1H)
10	44.6, d	1.69 (m, 1H)	38	27.9, t	1.34 (m, 2H)
11	72.3, d	3.84 (m, 1H)	39	30.2, t	1.34 (m, 2H)
12	33.6, t	1.61 (m, 1H); 1.32 (m, 1H)	40	30.4, t	1.32 (m, 2H)
13	30.7, t	1.28 (m, 2H)	41	30.6, t	1.42 (m, 1H); 1.26 (m, 1H)
14	40.8, d	1.59 (m, 1H)	42	27.7, t	1.35 (m, 2H)
15	72.2, d	3.90 (m, 1H)	43	29.9, t	1.57 (m, 2H)
16	41.8, t	1.77 (m, 1H); 1.65 (m, 1H)	44	42.4, t	3.15 (t, J = 7.1 Hz, 2H)
17	99.7, s	–	45	12.9, q	1.91 (s, 3H)
18	77.0, d	3.36 (d, J = 9.2 Hz, 1H)	46	17.1, q	1.10 (d, J = 6.6 Hz, 3H)
19	69.6, d	3.87 (m, 1H)	47	10.4, q	0.87 (d, J = 6.7 Hz, 3H)
20	41.2, t	1.89 (m, 1H); 1.30 (m, 1H)	48	14.7, q	0.90 (d, J = 6.5 Hz, 3H)
21	65.4, d	4.07 (m, 1H)	49	13.3, q	1.63 (s, 3H)
22	41.6, t	1.81 (m, 2H)	50	17.6, q	1.00 (d, J = 6.4 Hz, 3H)
23	70.6, d	5.24 (m, 1H)	51	14.4, q	0.94 (d, J = 6.6 Hz, 3H)
24	44.6, t	1.69 (m, 2H)	52	158.6, s	–
25	65.4, d	3.87 (m, 1H)	53	171.6, s	–
26	46.5, t	1.48 (m, 2H)	54	45.5, t	3.22 (s, 2H)
27	66.0, d	4.03 (m, 1H)	55	174.2, s	–
28	44.1, t	1.56 (m, 1H); 1.49 (m, 1H)			

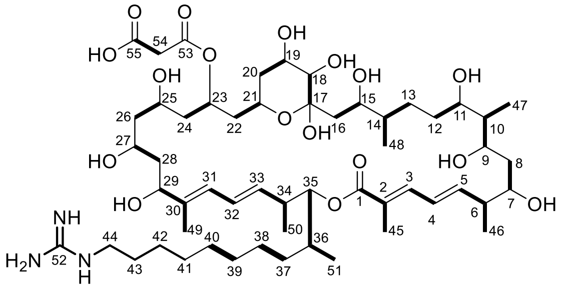

Supplementary Table 3. ^{1}H (600 MHz) and ^{13}C (150 MHz) NMR spectroscopic data (CH_3OH-d_4) of compound **3**.

	δ_C	δ_H		δ_C	δ_H
1	169.6, s	–	25	65.1, d	3.83 (m, 1H)
2	126.7, s	–	26	46.5, t	1.43 (m, 2H)
3	140.1, d	7.17 (d, J = 11.2 Hz, 1H)	27	65.5, d	3.81 (m, 1H)
4	127.6, d	6.44 (dd, J = 15.1, 11.3 Hz, 1H)	28	43.1, t	1.83 (m, 1H); 1.54 (m, 1H)
5	145.4, d	6.13 (dd, J = 15.2, 8.1 Hz, 1H)	29	77.5, d	5.42 (m, 1H)
6	43.9, d	2.49 (p, J = 7.5 Hz, 1H)	30	135.5, s	–
7	75.4, d	3.86 (m, 1H)	31	127.7, d	5.45 (m, 1H)
8	39.5, t	1.61 (m, 1H); 1.55 (m, 1H)	32	28.1, t	2.08 (m, 2H)
9	75.3, d	3.76 (m, 1H)	33	33.2, t	2.05 (m, 1H); 1.60 (m, 1H)
10	43.0, d	1.50 (m, 1H)	34	131.9, d	5.41 (m, 1H)
11	72.4, d	3.89 (m, 1H)	35	130.5, d	5.40 (m, 1H)
12	33.2, t	2.05 (m, 1H); 1.38 (m, 1H)	36	30.5, t	2.04 (m, 2H)
13	29.8, t	1.53 (m, 1H); 1.16 (m, 1H)	37	29.7, t	1.62 (m, 2H)
14	40.5, d	1.51 (m, 1H)	38	41.8, t	3.13 (t, J = 7.1 Hz, 2H)
15	72.3, d	3.79 (m, 1H)	39	12.9, q	1.93 (s, 3H)
16	42.5, t	2.06 (m, 1H); 1.64 (m, 1H)	40	16.4, q	1.13 (d, J = 6.8 Hz, 3H)
17	100.2, s	–	41	10.3, q	0.88 (d, J = 6.6 Hz, 3H)
18	78.5, d	3.19 (dd, J = 9.1, 4.1 Hz, 1H)	42	16.2, q	0.88 (d, J = 6.6 Hz, 3H)
19	69.5, d	3.83 (m, 1H)	43	12.6, q	1.61 (s, 3H)
20	40.8, t	1.90 (m, 1H); 1.28 (q, J = 11.8 Hz, 1H)	44	158.6, s	–
21	65.6, d	4.01 (t, J = 10.2 Hz, 1H)	45	171.8, s	–
22	42.1, t	1.77 (m, 1H); 1.63 (m, 1H)	46	45.6, t	3.21 (s, 2H)
23	70.2, d	5.26 (dt, J = 12.6, 6.5 Hz, 1H)	47	174.1, s	–
24	44.3, t	1.64 (m, 1H); 1.52 (m, 1H)			

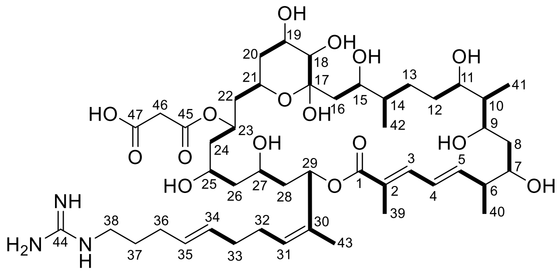

Supplementary Table 4. ^{1}H (600 MHz) and ^{13}C (150 MHz) NMR spectroscopic data (CH_3OH-d_4) of compound **4**.

	δ_C	δ_H		δ_C	δ_H
1	170.3, s	–	25	65.0, d	3.80 (m, 1H)
2	126.6, s	–	26	46.7, t	1.43 (m, 1H); 1.38 (m, 1H)
3	140.4, d	7.17 (d, J = 11.4 Hz, 1H)	27	65.3, d	3.69 (m, 1H)
4	127.7, d	6.45 (dd, J = 15.0, 11.3 Hz, 1H)	28	41.1, t	1.67 (m, 1H); 1.52 (m, 1H)
5	145.4, d	6.13 (dd, J = 15.1, 8.7 Hz, 1H)	29	75.6, d	5.24 (m, 1H)
6	44.2, d	2.47 (p, J = 6.8 Hz, 1H)	30	38.2, d	1.69 (m, 1H)
7	75.6, d	3.87 (m, 1H)	31	33.5, t	1.42 (m, 1H); 1.11 (m, 1H)
8	39.9, t	1.56 (m, 2H)	32	27.9, t	1.42 (m, 2H)
9	75.5, d	3.74 (m, 1H)	33	33.5, t	1.97 (m, 2H)
10	43.1, d	1.47 (m, 1H)	34	132.6, d	5.43 (dt, J = 15.0, 6.2 Hz, 1H)
11	72.3, d	3.90 (m, 1H)	35	130.2, d	5.40 (dt, J = 15.0, 6.2 Hz, 1H)
12	33.2, t	1.61 (m, 1H); 1.35 (m, 1H)	36	30.6, t	2.05 (m, 2H)
13	29.8, t	1.57 (m, 1H); 1.14 (m, 1H)	37	29.7, t	1.63 (m, 2H)
14	40.4, d	1.49 (m, 1H)	38	41.9, t	3.14 (m, 2H)
15	72.3, d	3.78 (m, 1H)	39	12.9, q	1.93 (s, 3H)
16	42.6, t	2.11 (m, 1H); 1.61 (m, 1H)	40	17.0, q	1.14 (d, J = 6.9 Hz, 3H)
17	100.3, s	–	41	10.2, q	0.88 (d, J = 6.8 Hz, 3H)
18	78.7, d	3.16 (m, 1H)	42	16.3, q	0.88 (d, J = 6.8 Hz, 3H)
19	69.6, d	3.82 (m, 1H)	43	15.3, q	0.92 (d, J = 6.8 Hz, 3H)
20	40.8, t	1.89 (dd, J = 10.8, 5.0 Hz, 1H);1.28 (q, J = 11.8 Hz, 1H)	44	158.2, s	–
21	65.6, d	4.00 (m, 1H)	45	171.8, s	–
22	42.1, t	1.75 (m, 1H); 1.61 (m, 1H)	46	45.5, t	3.21 (s, 2H)
23	70.0, d	5.27 (m, 1H)	47	174.0, s	–
24	44.5, t	1.62 (m, 1H); 1.50 (m, 1H)			

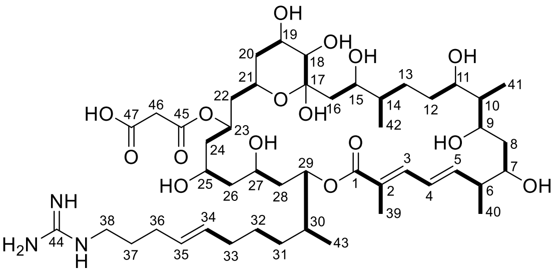

Supplementary Table 5. Bacterial strains used in this study.

Strains	Description	References
Escherichia coli		
DH10B	Host for general cloning	Invitrogen
ET12567/pUZ8002	Donor strain for conjugation between *E. coli* and *Streptomyces*	1
Streptomyces		
hygroscopicus LP-93	Polaramycin and rapamycin producer wild-type strain	2
211726	Azalomycin F producer wild-type strain	3,4
$\Delta ER_{1/2}/ER_{15}$	$ER_{1/2}$ replacement mutant with ER_{15} in 211726	This work
$\Delta ER_{1/2}$/pol-$ER_{1/2}$	$ER_{1/2}$ replacement mutant with pol-$ER_{1/2}$ in 211726	This work
$\Delta ER_{1/2}$/rap-ER_7	$ER_{1/2}$ replacement mutant with rap-ER_7 in 211726	This work
$\Delta ER_{1/2}$-$KR_{1/2}$/ER_{15}-KR_{15}	$ER_{1/2}$-$KR_{1/2}$ replacement mutant with ER_{15}-KR_{15} in 211726	This work
ΔazlB	*azlB* in-frame deletion mutant	This work
ΔazlB(ΔKS_6/KS_3)	KS_6 replacement mutant with KS_3 in ΔazlB	This work
ΔazlB(ΔAT_6/AT_3)	AT_6 replacement mutant with AT_3 in ΔazlB	This work
ΔazlB(ΔKA_6/KT_3)	KS_6-AT_6 replacement mutant with KS_3-AT_3 in ΔazlB	This work
ΔazlB(ΔACP_6/ACP_3)	ACP_6 replacement mutant with ACP_3 in ΔazlB	This work
ΔazlB(ΔKR_6/KR_3)	KR_6 replacement mutant with KR_3 in ΔazlB	This work
ΔazlB(ΔDH_6/DH_3)	DH_6 replacement mutant with DH_3 in ΔazlB	This work
ΔazlB(ΔDKA_6/DKA_3)	DH_6-KR_6-ACP_6 replacement mutant with DH_3-KR_3-ACP_3 in ΔazlB	This work

Supplementary Table 6. Plasmids used in this study.

Plasmids	Description	References
pYH7	*Streptomyces* - *E. coli* shuttle plasmid	5
pWHU5033	Plasmid for replacement of $ER_{1/2}$ with ER_{15} in vivo	This work
pWHU5034	Plasmid for replacement of $ER_{1/2}$ with pol-$ER_{1/2}$ in vivo	This work
pWHU5035	Plasmid for replacement of $ER_{1/2}$ with rap-ER_{7} in vivo	This work
pWHU5036	Plasmid for replacement of $ER_{1/2}$ -$KR_{1/2}$ with ER_{15}-KR_{15} in vivo	This work
pWHU5037	Plasmid for in-frame deletion of *azlB* in vivo	This work
pWHU5038	Plasmid for replacement of KS_{6} with KS_{3} in vivo	This work
pWHU5039	Plasmid for replacement of AT_{6} with AT_{3} in vivo	This work
pWHU5040	Plasmid for replacement of KS_{6}-AT_{6} with KS_{3}-AT_{3} in vivo	This work
pWHU5041	Plasmid for replacement of ACP_{6} with ACP_{3} in vivo	This work
pWHU5042	Plasmid for replacement of KR_{6} with KR_{3} in vivo	This work
pWHU5043	Plasmid for replacement of DH_{6} with DH_{3} in vivo	This work
pWHU5044	Plasmid for replacement of DH_{6}-KR_{6}-ACP_{6} with DH_{3}-KR_{3}-ACP_{3} in vivo	This work

Supplementary Table 7. Primers used in this study.

Primers	Oligonucleotide sequences (5' to 3')
**Primers for domain replacement vectors construction**	
ER_{15}-sw-L1	CGAATACTTCATATGCACTCCCCGCGGATGGACGC
ER_{15}-sw-L2	CATGTCCAGCCGCCACGCCGCGGTGCCGGCGGGGGCC
ER_{15}-sw-F	ACCGCGGCGTGGCGGCTGGACATGCACGCCAAGGGGA
ER_{15}-sw-R	CGGCATGGTCAGCGCCACCT
ER_{15}-sw-R1	TGGCGCTGACCATGCCGCACACCCTGAACCCCGAGGGT
ER_{15}-sw-R2	CAGGCATGCAAGCTTTACTGGTGGGAGAAGGGCAGC
pol-$ER_{1/2}$- sw-L1	CAAGGCGAATACTTCATATGCACTCCCCGCGGATGGACGC
pol-$ER_{1/2}$-sw-L2	GGTGCCGAGCTTCCACGCCGCGGTGCCGGCGGGGGCCTCG
pol-$ER_{1/2}$-sw-F	GCGTGGAAGCTCGGCACCACGGGC
pol-$ER_{1/2}$-sw-R	GTGTGGGACATGGTGAACACGTTCTTGCCG
pol-$ER_{1/2}$-sw-R1	GTGTTCACCATGTCCCACACCCTGAACCCCGAGGGTTCGG
pol-$ER_{1/2}$-sw-R2	ACCTGCAGGCATGCAAGCTTTACTGGTGGGAGAAGGGCAG
rap-ER_7- sw-L1	CAAGGCGAATACTTCATATGGGATGGACGCCATGCTGGAG
rap-ER_7-sw-L2	CTGCTCCAGCAGCCACGCCGCGGTGCCGGCGGGGG
rap-ER_7-sw-F	GCCGGCACCGCGGCGTGGCTGCTGGAGCAGTCCCACAGCG
rap-ER_7-sw-R	GGGGTTCAGGGTGTGAGGGACGGTGAGGACGATCT
rap-ER_7-sw-R1	CGTCCCTCACACCCTGAACCCCGAGGGT
rap-ER_7-sw-R2	ACCTGCAGGCATGCAAGCTTTACTGGTGGGAGAAGGGCAG
EK_{15}-sw-L1	CGAATACTTCATATGAGCGGGTGGCGTTCCTCTTCAC
EK_{15}-sw-L2	GGTCCAGTCCAGCGTGAACAGCGTTCCGTCGCCAC
EK_{15}-sw-F	ACGCTGTTCACGCTGGACTGGACCCCCGTACC
EK_{15}-sw-R	CACCCGGACCACACCGCGCAGCAGCGCGGGTACCGGC
EK_{15}-sw-R1	CTGCGCGGTGTGGTCCGGGTGCGGAGCAAGGCGGCGG
EK_{15}-sw-R2	CAGGCATGCAAGCTTAGGTGATCGGGTCCCGCCAGTG
ΔazlB-L1	GAATACTTCATATGTATCGGGCCGACGGCGAAGCT
ΔazlB-L2	GGTCGCCGAGCTGATCAGCTGGCG

ΔazlB-R1	ATCAGCTCGGCGACCGAACCCATCGCCATCGTCGG
ΔazlB-R2	GCAGGCATGCAAGCTTCAGGCCGACCGACACCATG
KT_3-sw-L1	CAAGGCGAATACTTCATATG CCTTCAGCCGTCAGCGCGGA
KT_3-sw-L2	TCGCGCTGGAAGACATACGTCGGCAGCTCGACGCGGCGGG
KT_3-sw-R1	CGCGTCGAGCTGCCGACGTATGTCTTCCAGCGCGAGCGGT
KT_3-sw-R2	ACCTGCAGGCATGCAAGCTTCGCAGATCCCGCTCGCCGAG
DKA_3-sw-L1	CAAGGCGAATACTTCATATGCGACGCCGGGCGTGTTCATC
DKA_3-sw-L2	GCGTAGGTCGGCAGCTCGACCCGTCGG
DKA_3-sw-F	GTCGAGCTGCCGACCTACGCCTTCCAGCACGA
DKA_3-sw-R	GGCGCTCTCGCCGCCAAGGATCTCCGAGCGCAGATAG
DKA_3-sw-R1	TCGGAGATCCTTGGCGGCGAGAGCGCCACCTCGTCGG
DKA_3-sw-R2	CAGGCATGCAAGCTTTCCAGGGAGAAGACCCCCGCCA
KS_3-sw-L1	CGAATACTTCATATGATCGGGCCGACGGCGAAGCT
KS_3-sw-L2	TACCGATGATGGCAATGGGTTCGGTCGCCGAGCTGATCAGC
KS_3-sw-F	GAACCCATTGCCATCATCGGTAT
KS_3-sw-R	GGTATCGCGGGCCCGGTGGCGTGG
KS_3-sw-R1	TCGCGGGCCCGGTGGCGTGGGTGGTGTCGGGCCATGGCGC
KS_3-sw-R2	CAGGCATGCAAGCTTCCACCCGCCAGCCCCTCACTCA
AT_3-sw-L1	CGAATACTTCATATGGGTGCGATCCGCATCGAAGAAG
AT_3-sw-L2	ACAAACACCGAACGACCGGGAGGCGCGC
AT_3-sw-F	CGGTCGTTCGGTGTTTGTGTTCCCGGGGCAGG
AT_3-sw-R	ACACAAACACCGAACGACCGGGAGGCGCGC
AT_3-sw-R1	CGGTCGTTCGGTGTTTGTGTTCCCGGGGCAGG
AT_3-sw-R2	CAGGCATGCAAGCTTCCCCACGCCATCGACACACCCG
KR_3-sw-L1	CAAGGCGAATACTTCATATGGCGCGCCTCCCGGTC
KR_3-sw-L2	GTCCACCCACTCCAGCCGGAACAACCCATCCCGCG
KR_3-sw-F	GATGGGTTGTTCCGGCTGGAGTGGGTGGACGCGCC
KR_3-sw-R	TTCGGCGCCCTGCGCACGCAGCGCCGGGATGTCCA
KR_3-sw-R1	ATCCCGGCGCTGCGTGCGCAGGGCGCCGAACTCCC

KR_3-sw-R2	ACCTGCAGGCATGCAAGCTTGCGACCTCTCCGGCC
DH_3-sw-L1	CGAATACTTCATATGGACGCTGCAAGTCCTTCTCGAA
DH_3-sw-L2	CCCACATCGCCCGCGTCGGCGGGAGCCTCGAGCCAG
DH_3-sw-F	CCCGCCGACGCGGGCGATGTGGGTTCGGTCGGGCTGG
DH_3-sw-R	CTGACCCGCATCAACCGGACGCAACACCAACGACTCA
DH_3-sw-R1	TGCGTCCGGTTGATGCGGGTCAGCTGAATGCT
DH_3-sw-R2	CAGGCATGCAAGCTTCCCCGCTTGGTCACGGGGTCGA
ACP_3-sw-L1	CAAGGCGAATACTTCATATGTCGAGTTGCTGGTGCAGGCG
ACP_3-sw-L2	CTGAGCAGTCGCCGCCGCAGTTCGTCGGGAAGGCTTTCGC
ACP_3-sw-F	GCCTTCCCGACGAACTGCGGCGGCGACTGCTCAGCATGTC
ACP_3-sw-R	GGTGGCGCTCTCGCCGCCCAGGATCTCCGAGCGCAGATAG
ACP_3-sw-R1	TGCGCTCGGAGATCCTGGGCGGCGAGAGCGCCACCTCGTC
ACP_3-sw-R2	ACCTGCAGGCATGCAAGCTTAGGGAGAAGACCCCCGCCAC
**Primers for mutant verification**	
ER_{15}-sw-CK-L1	ACGGGTCCGGGCGAGGGCGTCG
ER_{15}-sw-CK-L2	ACGTCCAGTCCCGCGGGACCCG
ER_{15}-sw-CK-R1	AGATGGGCAAGACCGACATCAGGA
ER_{15}-sw-CK-R2	TCGATCCGCTTCGGCGACAGGGT
pol-sw-CK-L1	ACGTCGAGAACCTCGCCGAG
pol-sw-CK-L2	GGCCCGAACGATGCGTTGATCA
pol-sw-CK-R1	AGTTCGAGCGACGGTTCCTGGA
pol-sw-CK-R2	TCACGAACTCCGCCAGGTCCAT
rap-sw-CK-L1	ACGTCGAGAACCTCGCCGAG
rap-sw-CK-L2	CACCAGCCCGTAATACGCGGTG
rap-sw-CK-R1	TCGTCCTGAACTCCCTCAGCGG
rap-sw-CK-R2	TCACGAACTCCGCCAGGTCCAT
EK_{15}-sw-CK-L1	CGCTCTGCACGCCATGGAGAT
EK_{15}-sw-CK-L2	CTGGATCATGCCGAGCGTGTGG

EK_{15}-sw-CK-R1	ATCTCGACCTGGCCGCCTTCAT
EK_{15}-sw-CK-R2	GGGCGTGGGGTTGTCGAAGATC
ΔazlB-CK-F	TTGTCAACCATGCATGGATGGACTT
ΔazlB-CK-R	AGGTCTCCAGCAGCAGCCGCTGCTG
KT_3-sw-CK-L	CGCACTCTTCGCATGTGGAGGG
KT_3-sw-CK-R	GCCTCACCCACCTCGACCTGAA
DKA_3-sw-CK-L1	GGTCTGGACGCGGAGTACTGGT
DKA_3-sw-CK-L2	AACCGAGCACACCATGAGCGTG
DKA_3-sw-CK-R1	GCCCTCTTCCGCAACGTCGTAC
DKA_3-sw-CK-R2	ATGTCGAAGAAGGCGGGGTCGA
KS_3-sw-CK-L1	ACAGCGCGTGAAATTTCGGGGA
KS_3-sw-CK-L2	AGGTACGGCTCGATCTCGTCCG
KS_3-sw-CK-R1	GTCGAACCTCGGCCACACACAG
KS_3-sw-CK-R2	CACATCCACCCGGTCAAACCCG
AT_3-sw-CK-L1	GCATGTGATCCTGGAGCAGGCC
AT_3-sw-CK-L2	CCGCGATCTCACCCTGACTGTG
AT_3-sw-CK-R1	CGCACTCTTCGCATGTGGAGGG
AT_3-sw-CK-R2	TGCACCAGCAACTCGACGAACG
DH_3-sw-CK-L1	GTGTTCATCGAGGTGAGCCCGC
DH_3-sw-CK-L2	AACCGAGCACACCATGAGCGTG
DH_3-sw-CK-R1	GACTTTGGTGTGTGGCCTCCGG
DH_3-sw-CK-R2	AGCACCAGACGCCCAGGATTCT
KR_3-sw-CK-L1	TGGTCTGCATCCGGCGTTGTTC
KR_3-sw-CK-L2	AGCACCAGACGCCCAGGATTCT
KR_3-sw-CK-R1	CTGTCGGCGTTCGTGCTGTTCT
KR_3-sw-CK-R2	CCATCGGGGTCGGGTAGTCGAA
ACP_3-sw-CK-L	TGTCGATGGCGTGGGGTCTGT
ACP_3-sw-CK-R	ATGTCGAAGAAGGCGGGGTCGA

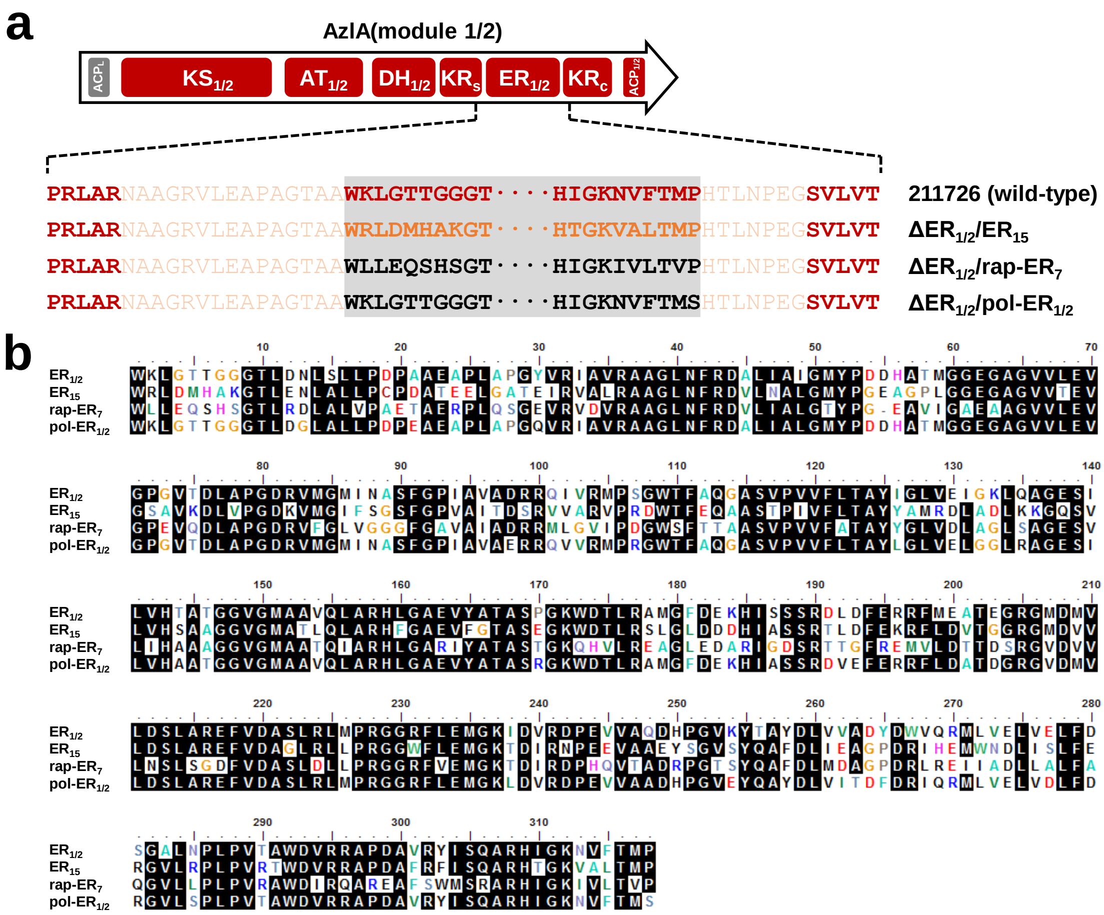

Supplementary Fig. 1. $ER_{1/2}$ domain replacements based on wild-type strain. (**a**) The ER domains used for replacement were highlighted in gray. The link region between domains were shown in non-bold letters. (**b**) The multiple sequence alignment of ER domains from module 1/2 of azalomycin ($ER_{1/2}$), module 7 of rapamycin (rap-ER_7) and module 1/2 of polaramycin (pol-$ER_{1/2}$) biosynthetic gene cluster, respectively. Source data are provided as a Source Data file.

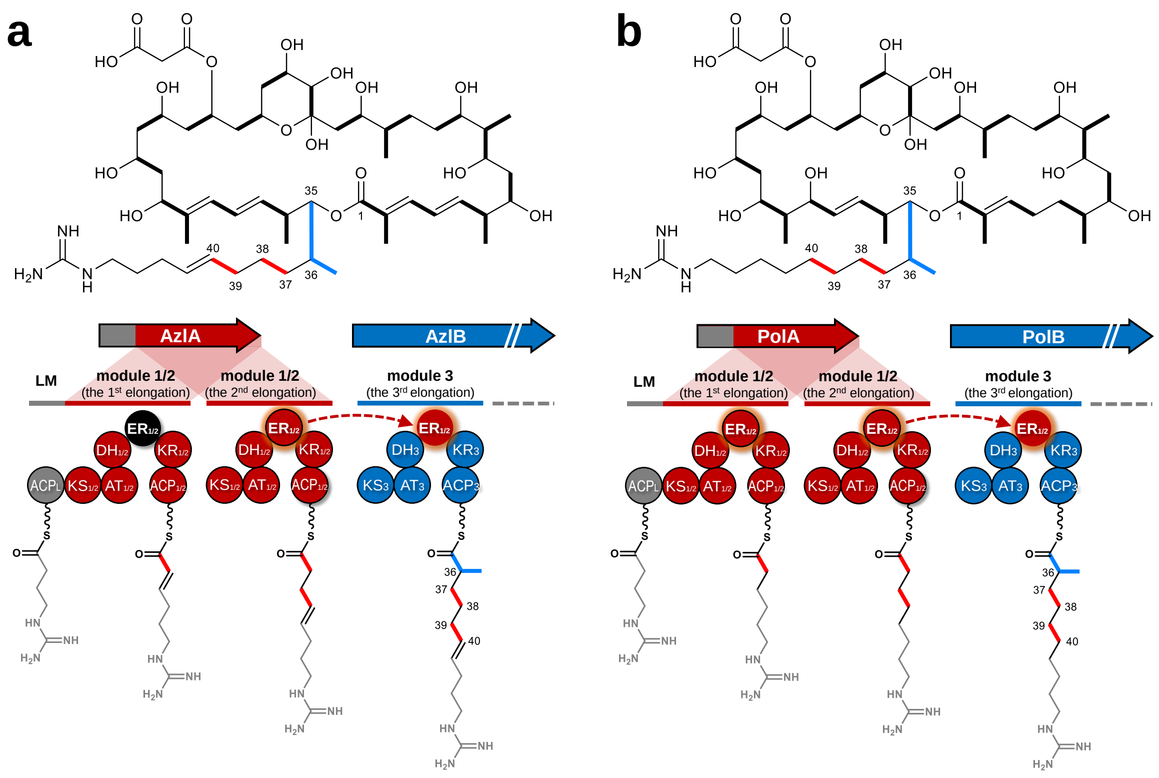

Supplementary Fig. 2. Comparison of structure and non-colinear enoylreduction between AZL F3a (a) and polaramycin A (b) PKS. The cross-module enoylreduction in module 3 supplied by $ER_{1/2}$ is indicated by dotted arc arrows.

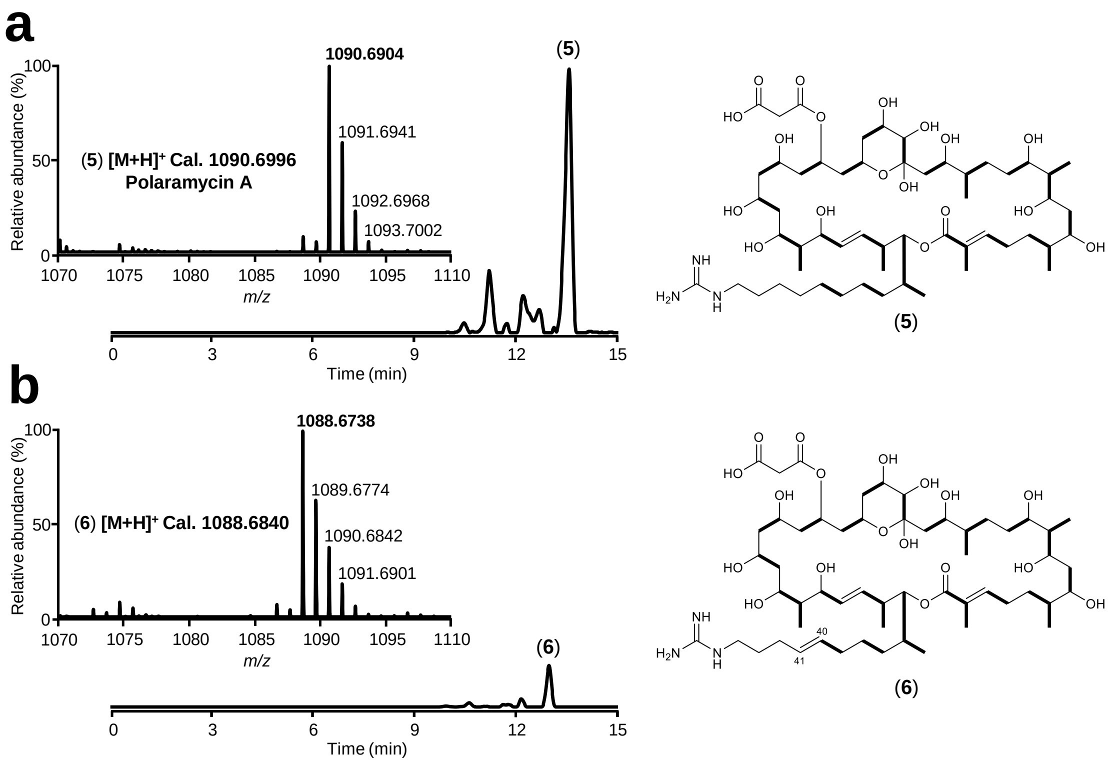

Supplementary Fig. 3. LC-ESI-HRMS analysis of polaramycin A (a) and its unsaturated analog (b) produced by *S. hygroscopicus* LP-93. The spectra were extracted at *m/z* ($[M+H]^+$) 1090.6996 and 1088.6840 corresponding to polaramycin A (**5**) and its unsaturated analog (**6**) with double bond at C40-C41, respectively.

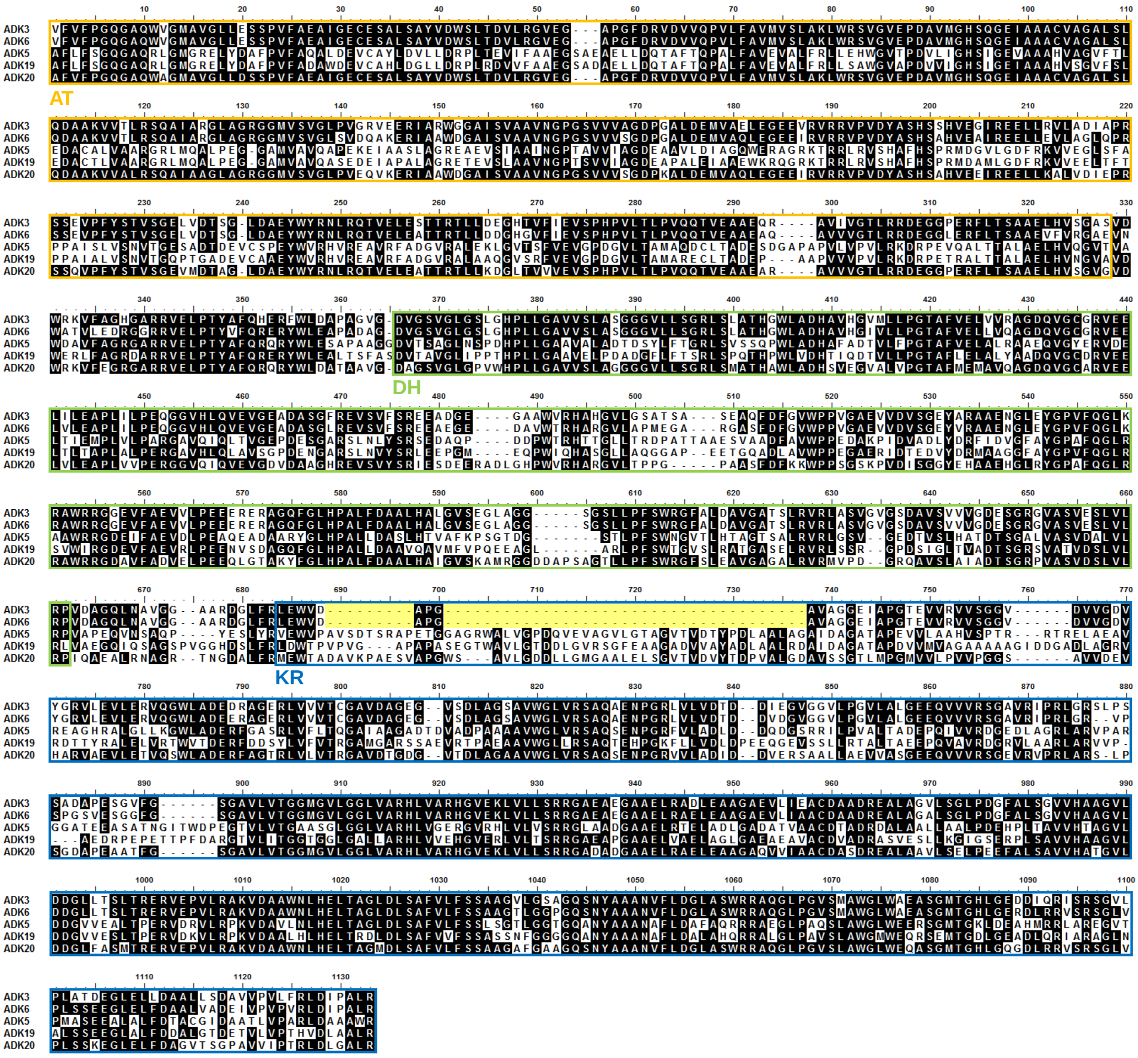

Supplementary Fig. 4. Alignment of AT-DH-KR tridomains from modules 3, 5, 6, 19 and 20 that contain 5 domains. The domains are indicated with color boxes. The apparent difference of 46 amino acids absence in KR_3 and KR_6 are highlighted in yellow.

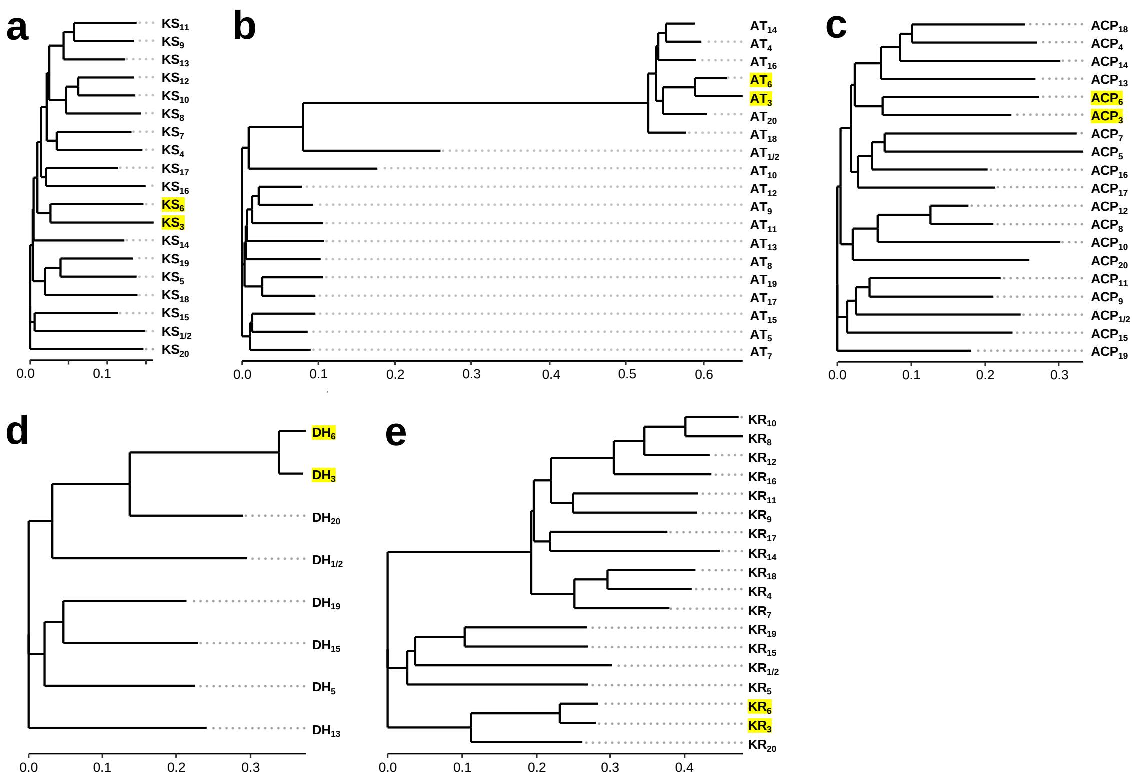

Supplementary Fig. 5. Domain phylogenetic analysis of AZL PKS. KS (**a**), AT (**b**), ACP (**c**), DH (**d**) and KR (**e**) domains from module 3 and 6 were highlighted in yellow.

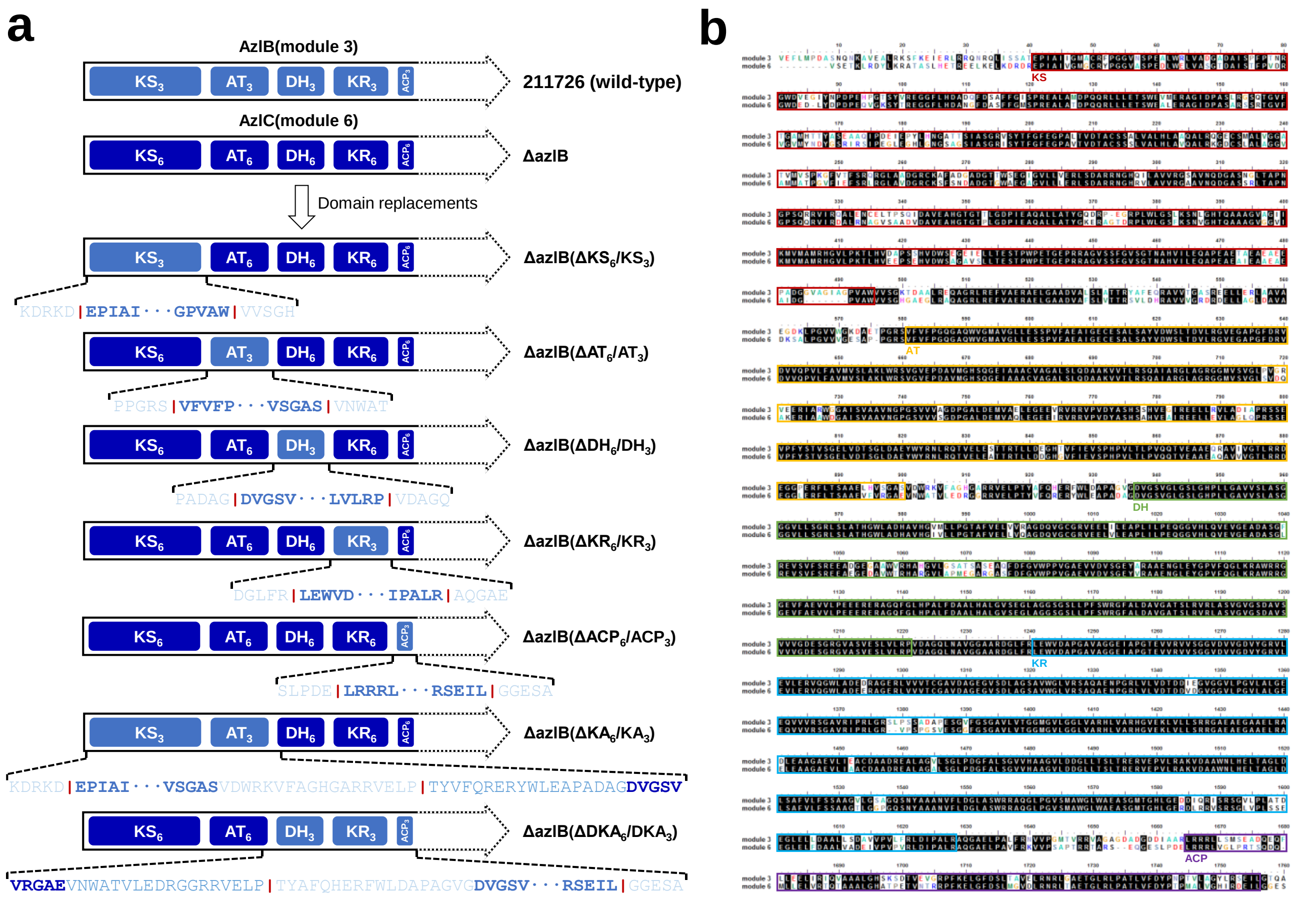

Supplementary Fig. 6. Domain replacements based on ΔazIB mutant. (**a**) The representation of domain replacements. The domains and link region between domains in enlarged regions are shown in bold and non-bold letters, respectively, in which the red short vertical lines indicate the joint sites after replacement. (**b**) The sequence alignment of module 3 and 6. The domains are indicated with color boxes. Source data are provided as a Source Data file.

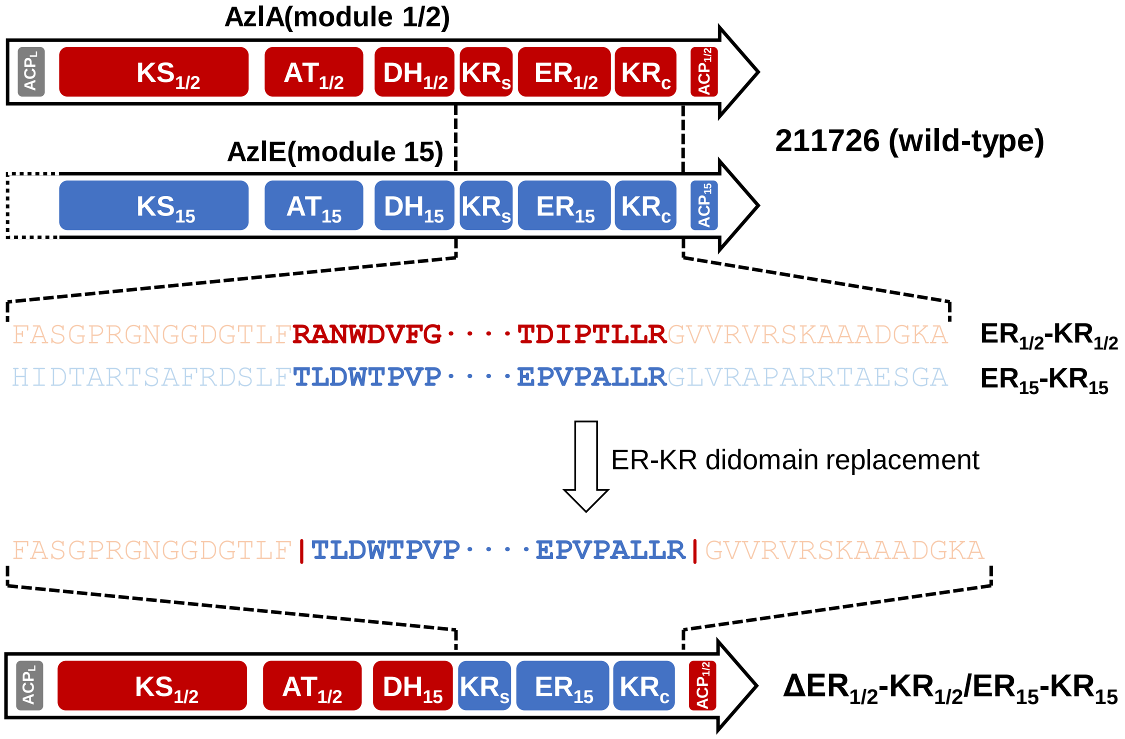

Supplementary Fig. 7. $ER_{1/2}$-$KR_{1/2}$ didomain replacement with ER_{15}-KR_{15} based on wild-type strain. The domains and link region between domains in enlarged regions are shown in bold and non-bold letters, respectively, in which the red short vertical lines indicate the joint sites after replacement. Source data are provided as a Source Data file.

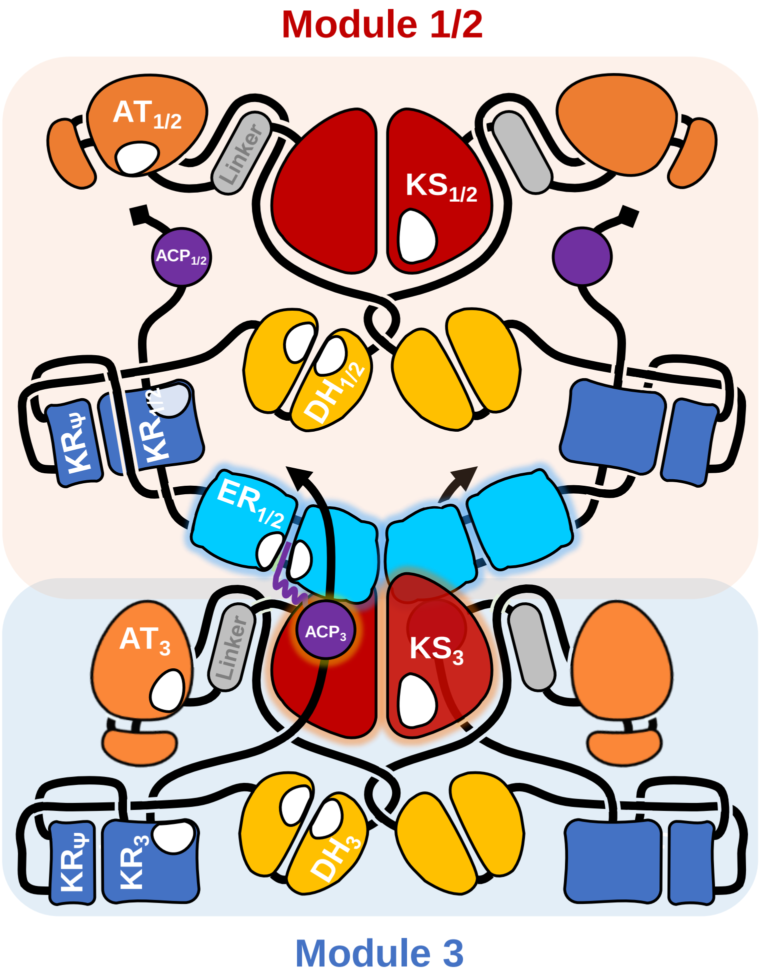

Supplementary Fig. 8. The proposed domain topologies of module 1/2 and module 3 in the state of cross-module enoylreduction. In the process of cross-module enoylreduction, a certain angle of clockwise rotation along the X axis of KR$_{1/2}$ and ER$_{1/2}$ facilitated the connection between ER$_{1/2}$ and KS$_{3}$ with the substrate tunnel orientation of ER$_{1/2}$ toward the reaction chamber of module 3, which made it accessible for the intermediate tethered onto ACP$_{3}$ to be reduced.

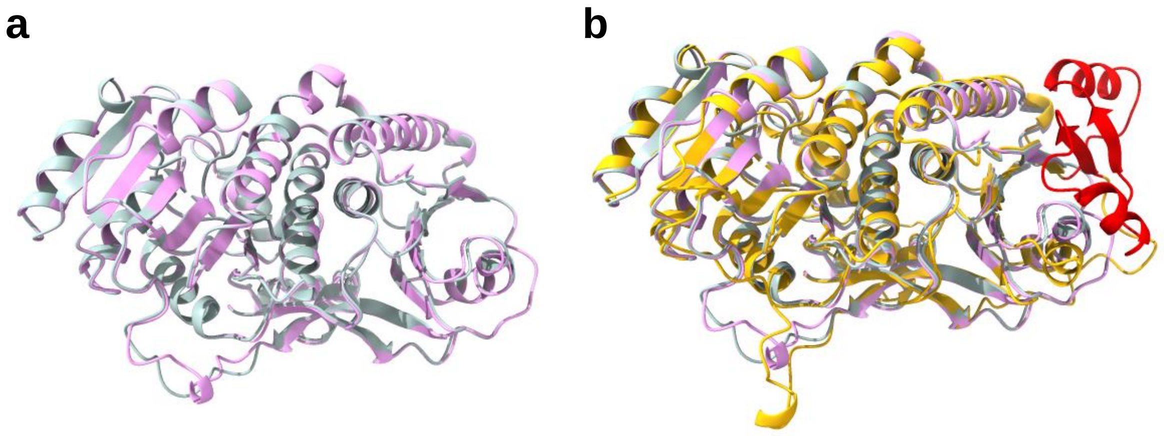

Supplementary Fig. 9. The structural alignment of KR domains. (**a**) The structural alignment between KR_3 (pale purple), and KR_6 (light cyan) show a highly structural coincidence. (**b**) The structural alignment among KR_3 (pale purple), KR_6 (light cyan), and KR_5 (yellow) reveal a truncated structural subdomain in KR_3 and KR_6, where two α-helixes and two β-folds (red) existing only in KR_5 are missing. These KR structures are obtained using AlphaFold[6].

Supplementary Note 1. NMR spectra of compound **1**.

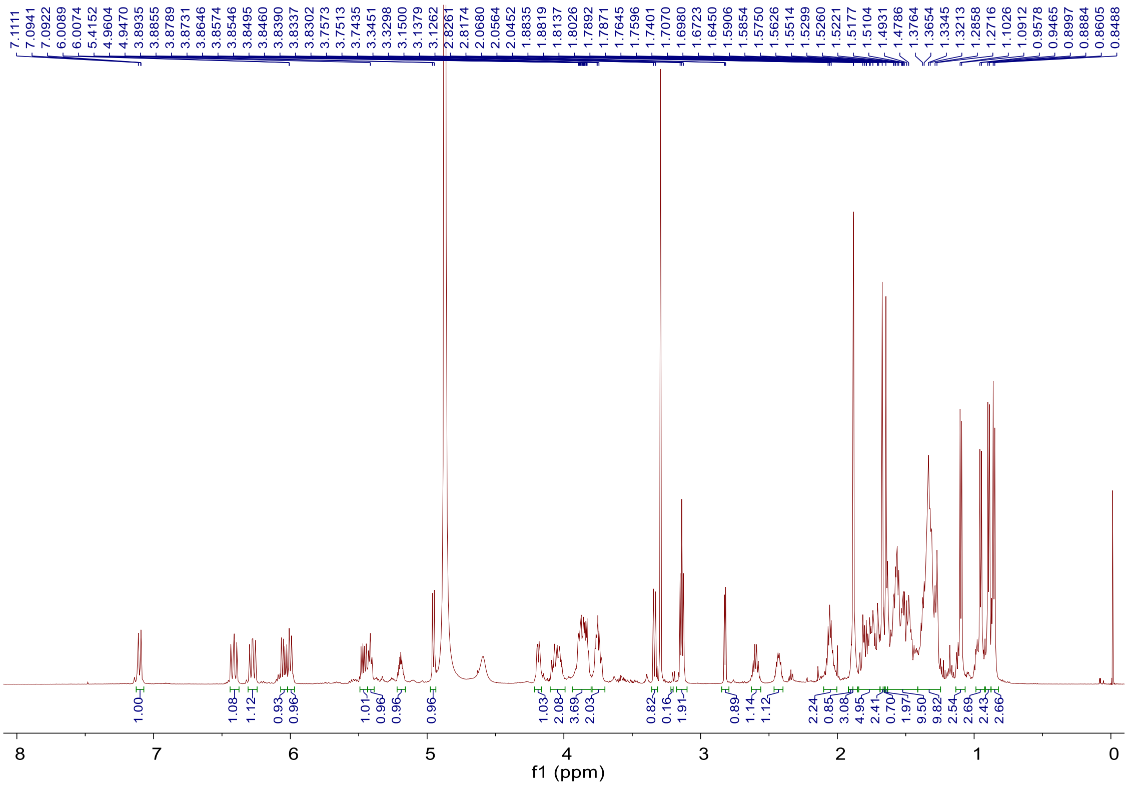

Supplementary Fig. 10. ^{1}H NMR spectrum (600 MHz, CD_3OD) of compound **1**.

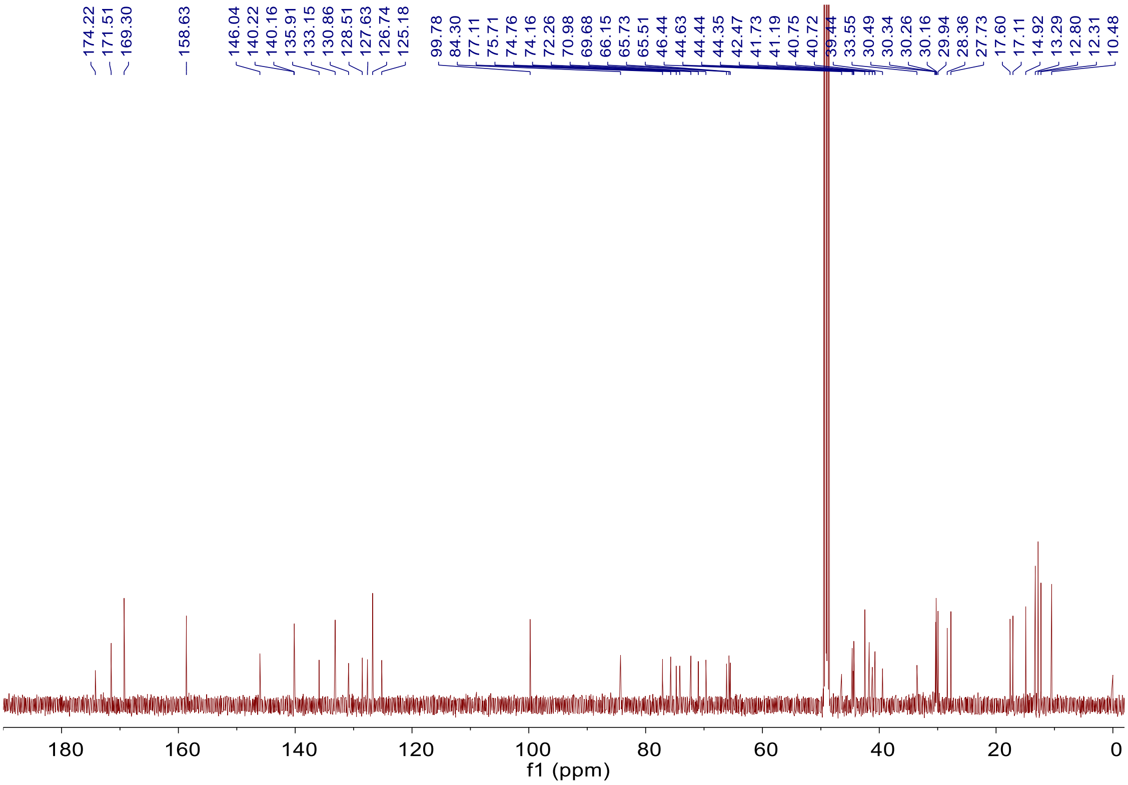

Supplementary Fig. 11. ^{13}C NMR spectrum (150 MHz, CD_3OD) of compound **1**.

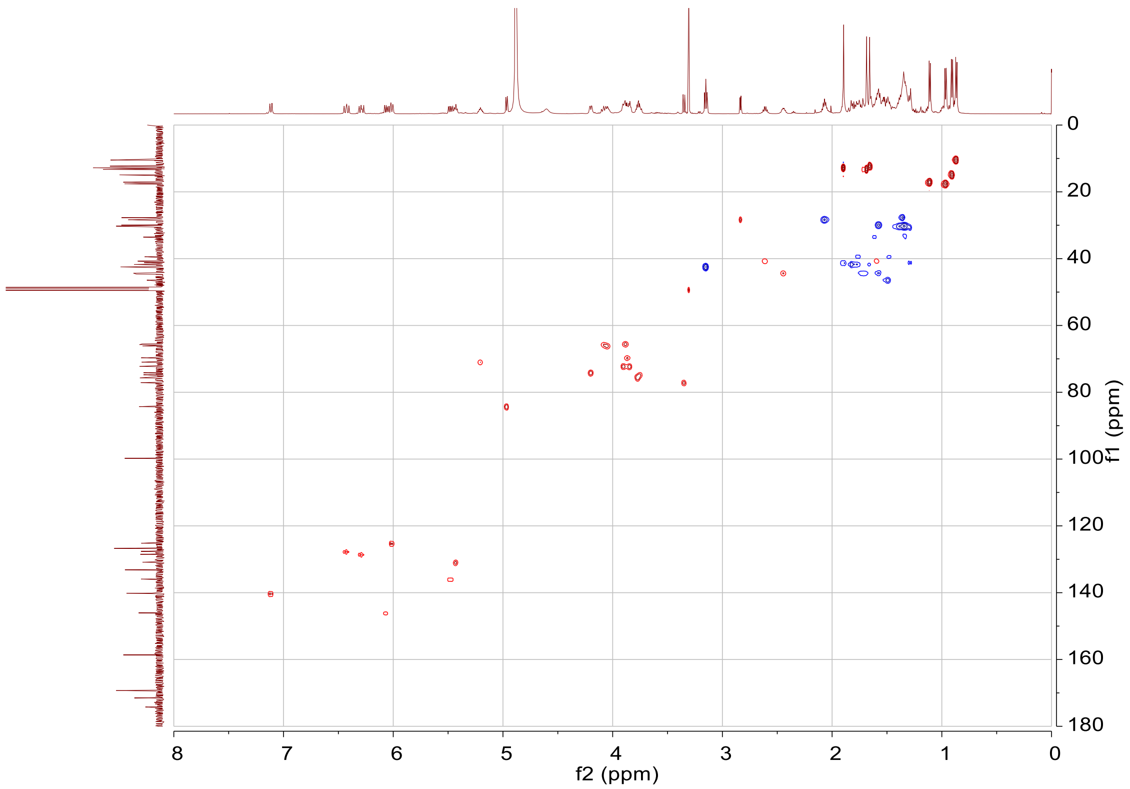

Supplementary Fig. 12. HSQC spectrum (600 MHz, CD_3OD) of compound **1**.

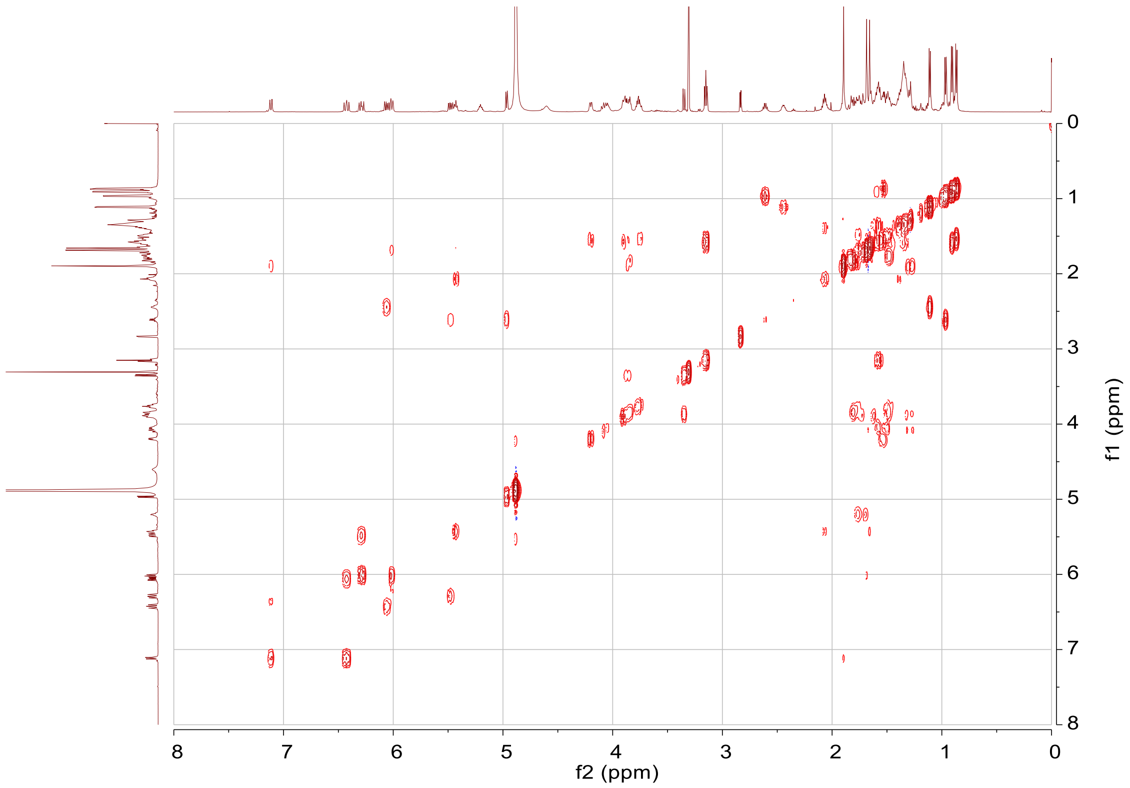

Supplementary Fig. 13. ^{1}H-^{1}H COSY spectrum (600 MHz, CD_3OD) of compound **1**.

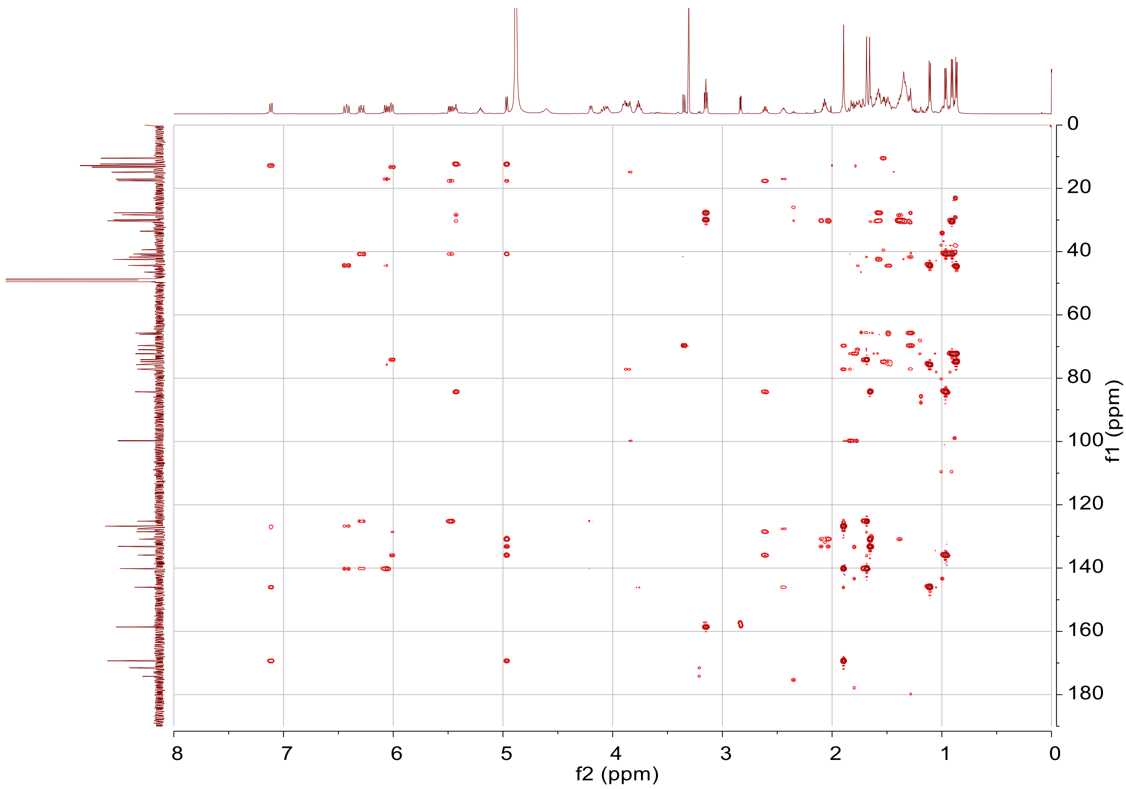

Supplementary Fig. 14. HMBC spectrum (600 MHz, CD_3OD) of compound **1**.

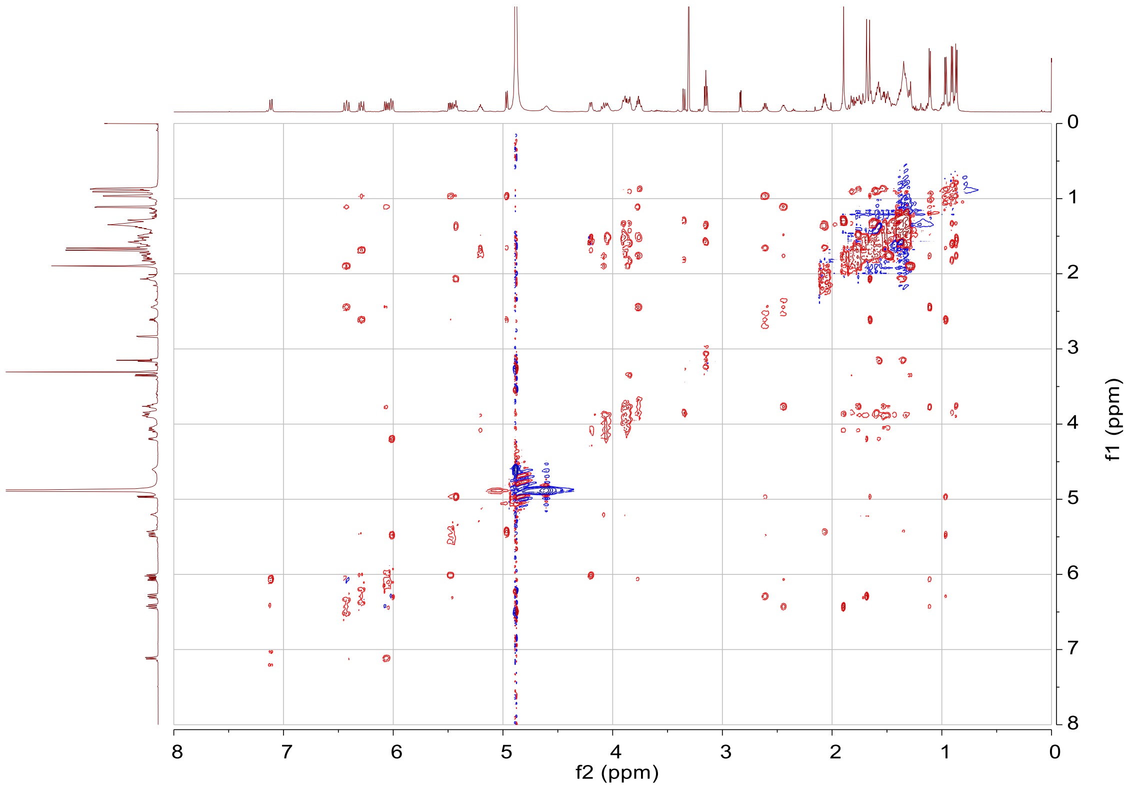

Supplementary Fig. 15. ROESY spectrum (600 MHz, CD_3OD) of compound **1**.

Supplementary Note 2. NMR spectra of compound **2**.

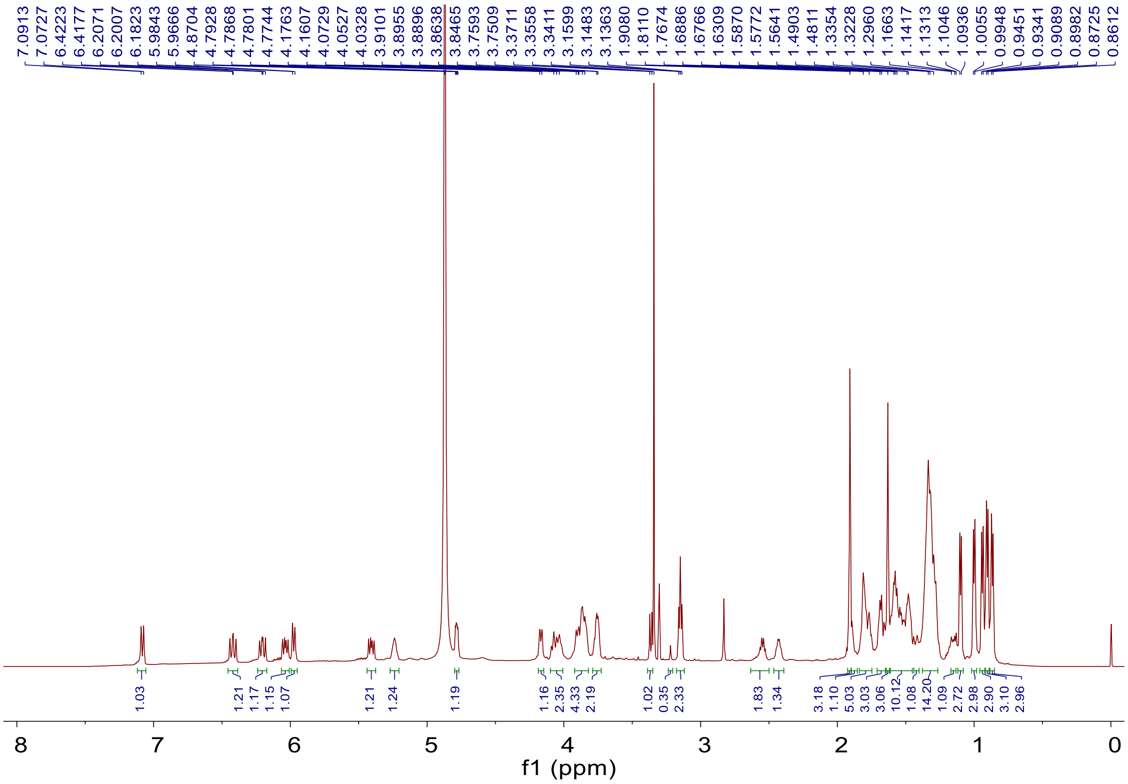

Supplementary Fig. 16. ^{1}H NMR spectrum (600 MHz, CD_3OD) of compound **2**.

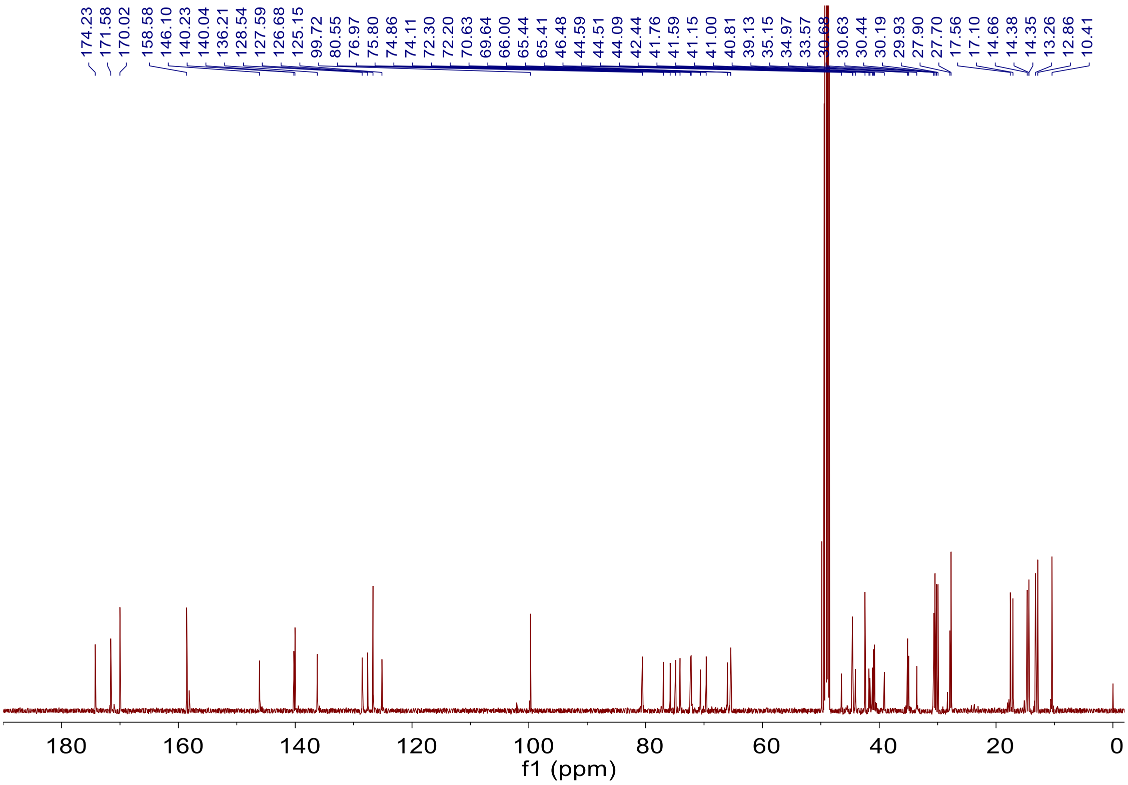

Supplementary Fig. 17. ^{13}C NMR spectrum (150 MHz, CD_3OD) of compound **2**.

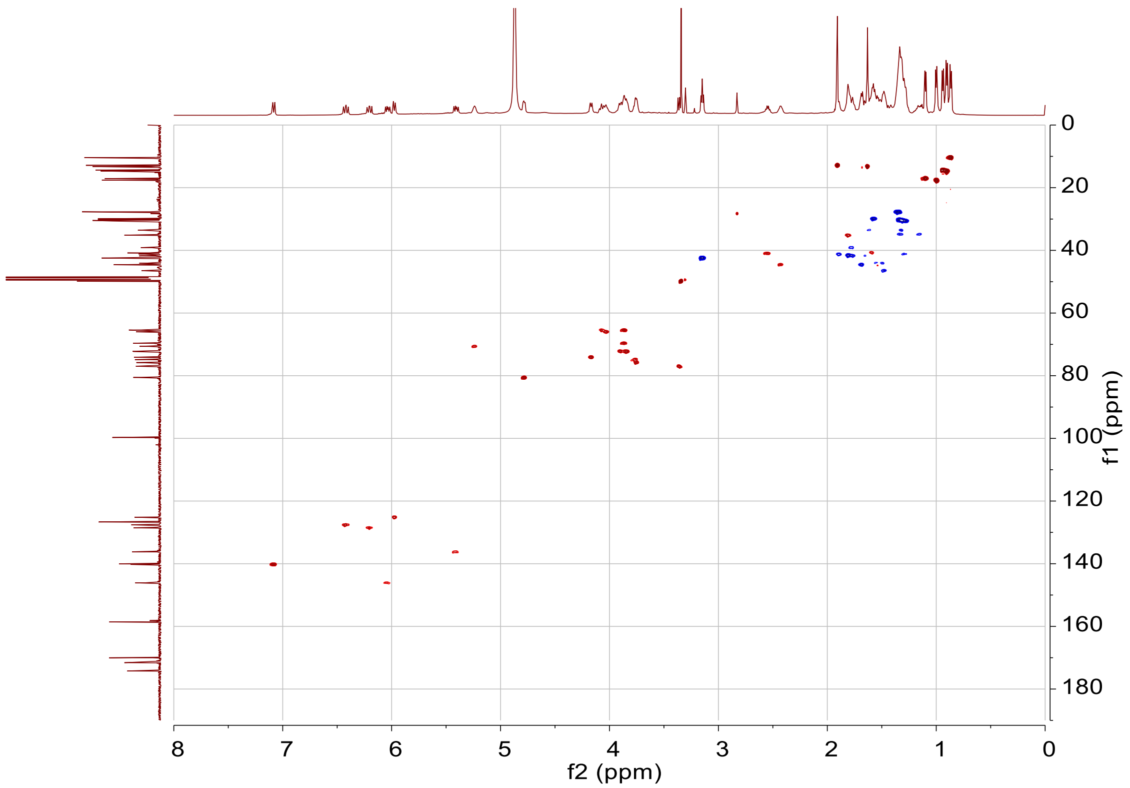

Supplementary Fig. 18. HSQC spectrum (600 MHz, CD_3OD) of compound **2**.

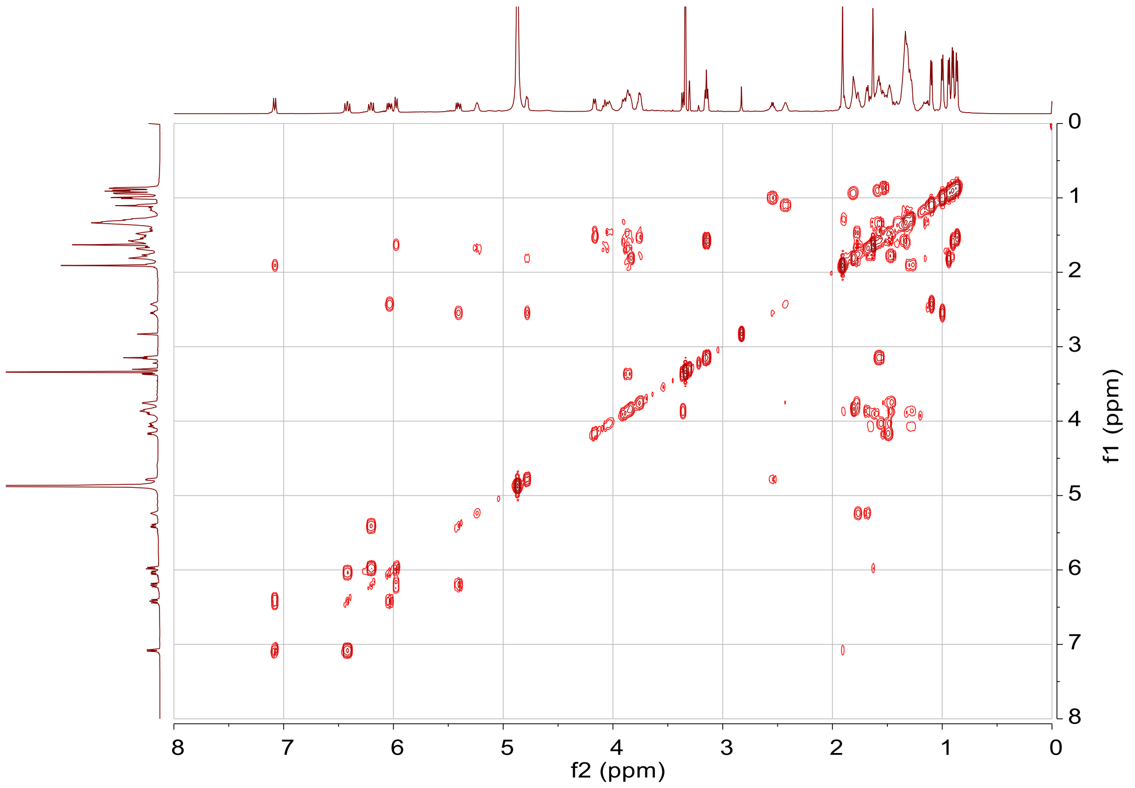

Supplementary Fig. 19. ^1H-^1H COSY spectrum (600 MHz, CD_3OD) of compound **2**.

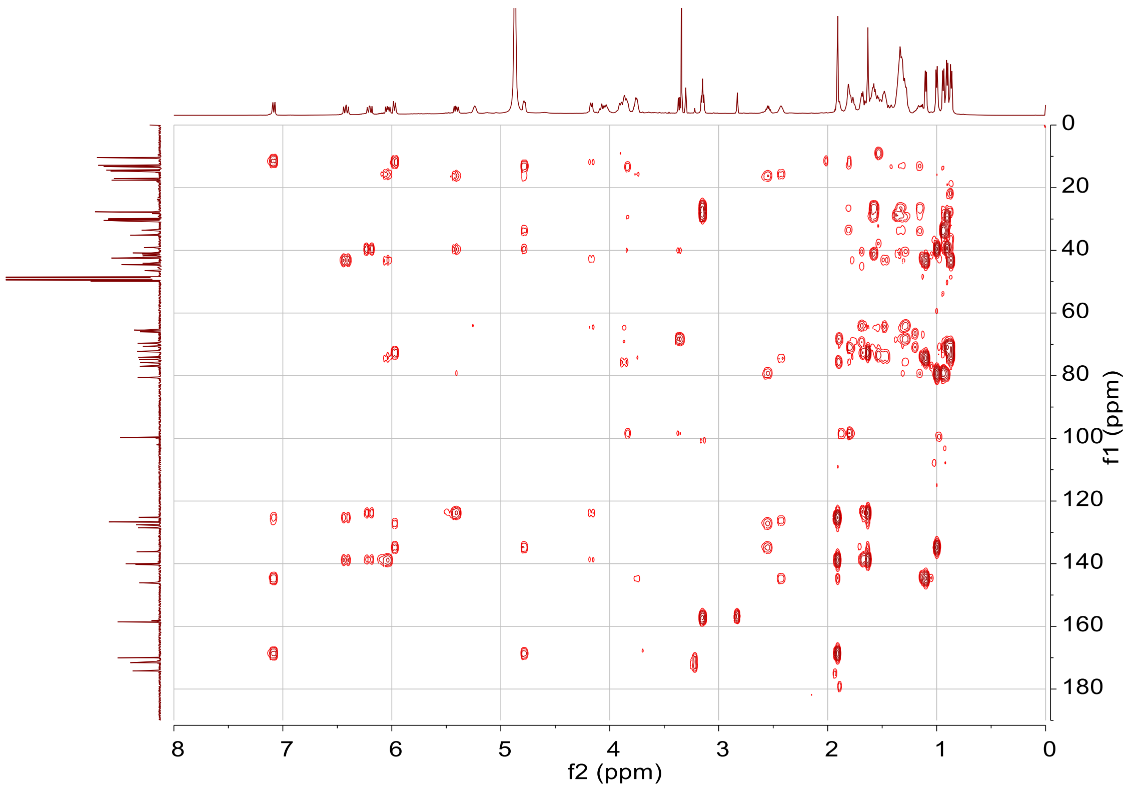

Supplementary Fig. 20. HMBC spectrum (600 MHz, CD_3OD) of compound **2**.

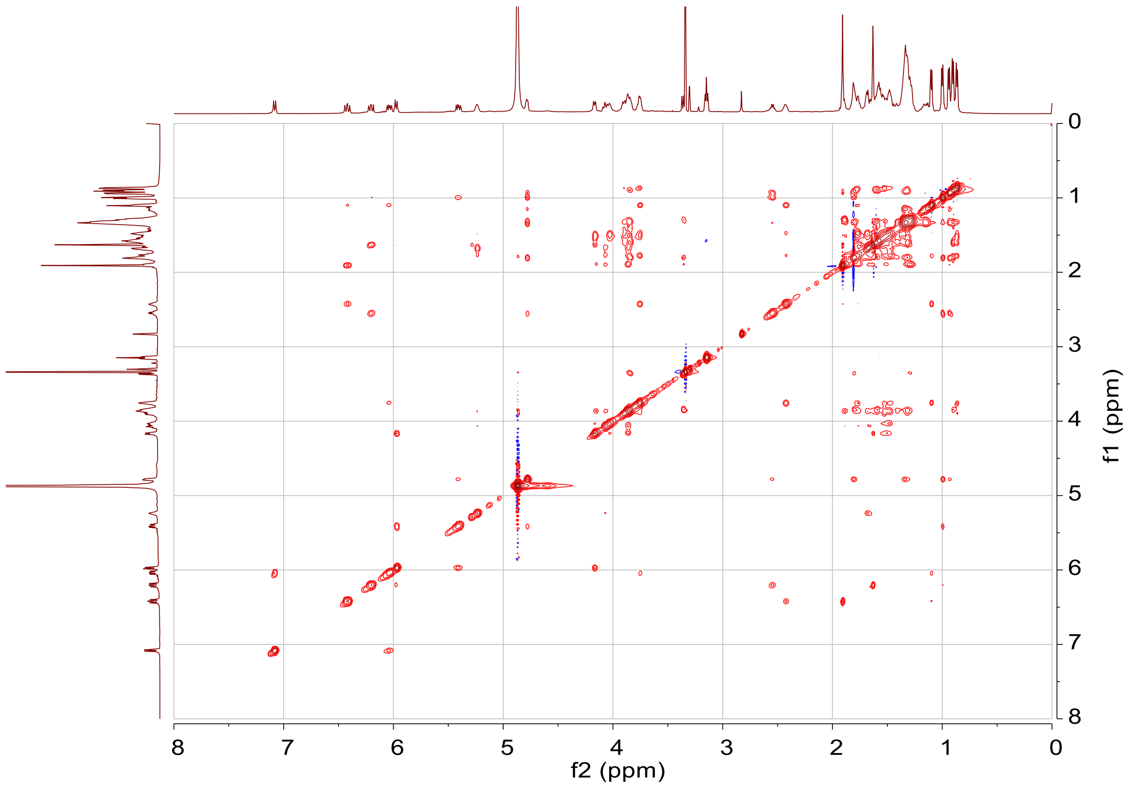

Supplementary Fig. 21. NOESY spectrum (600 MHz, CD_3OD) of compound **2**.

Supplementary Note 3. NMR spectra of compound **3**.

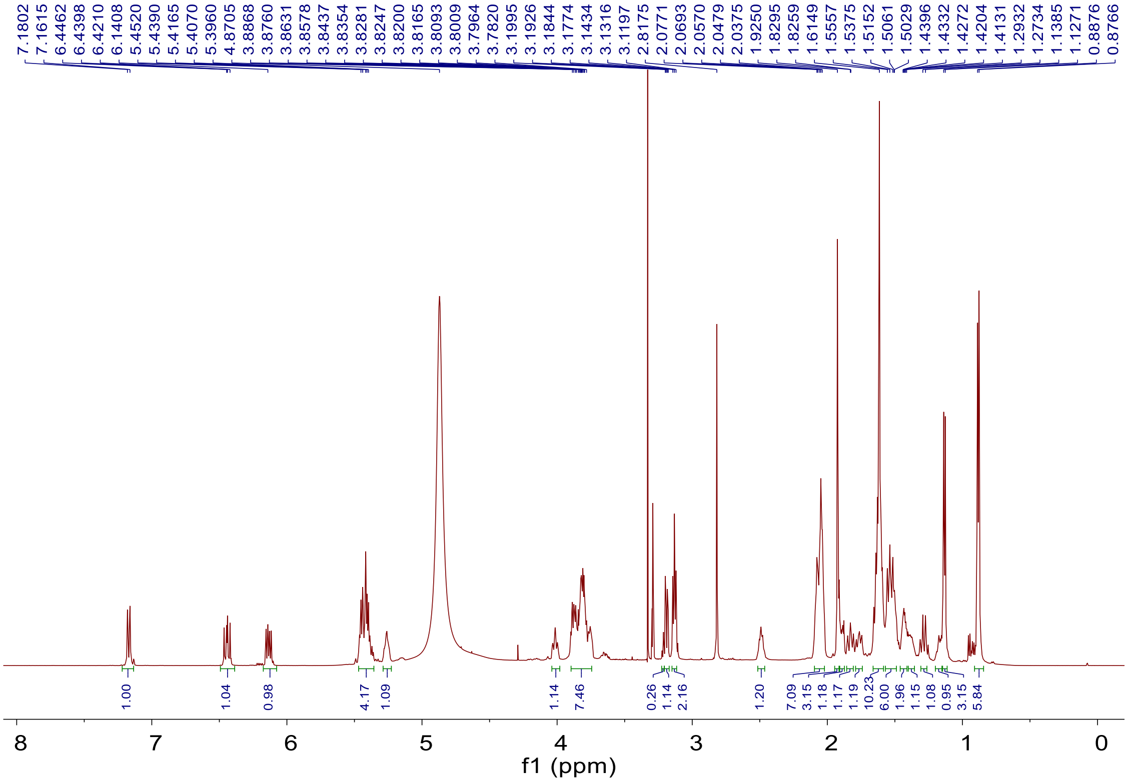

Supplementary Fig. 22. ^{1}H NMR spectrum (600 MHz, CD_3OD) of compound **3**.

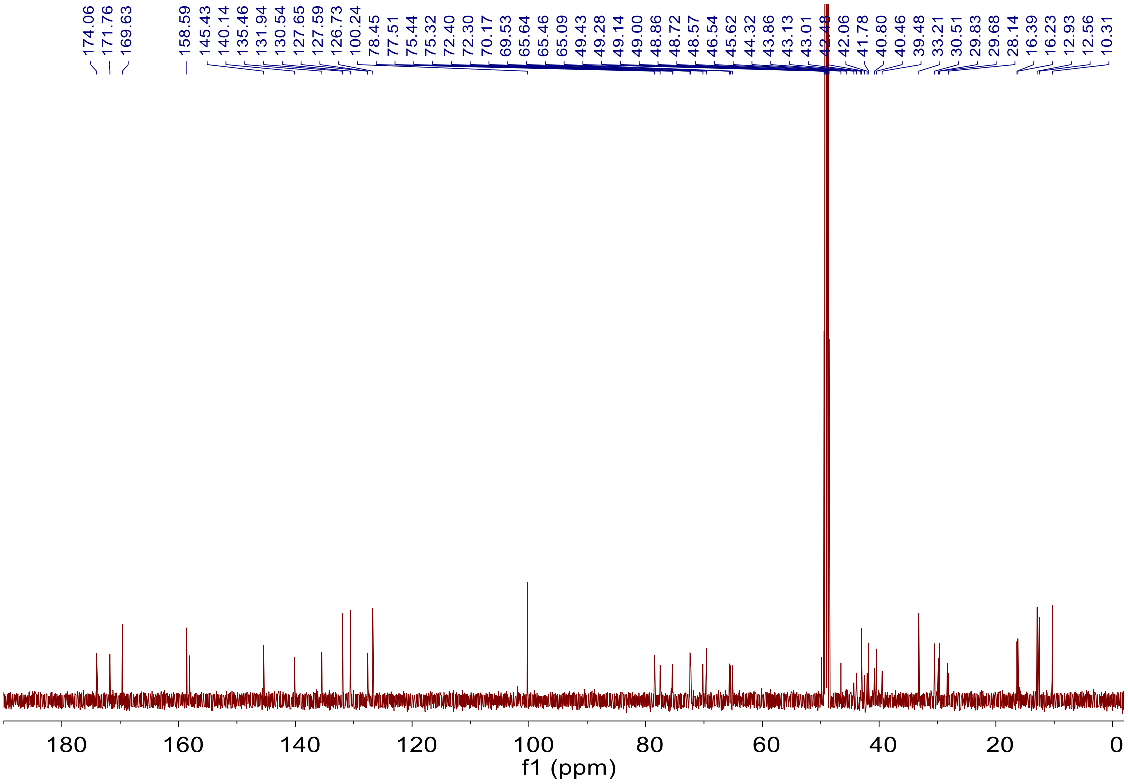

Supplementary Fig. 23. ^{13}C NMR spectrum (150 MHz, CD_3OD) of compound **3**.

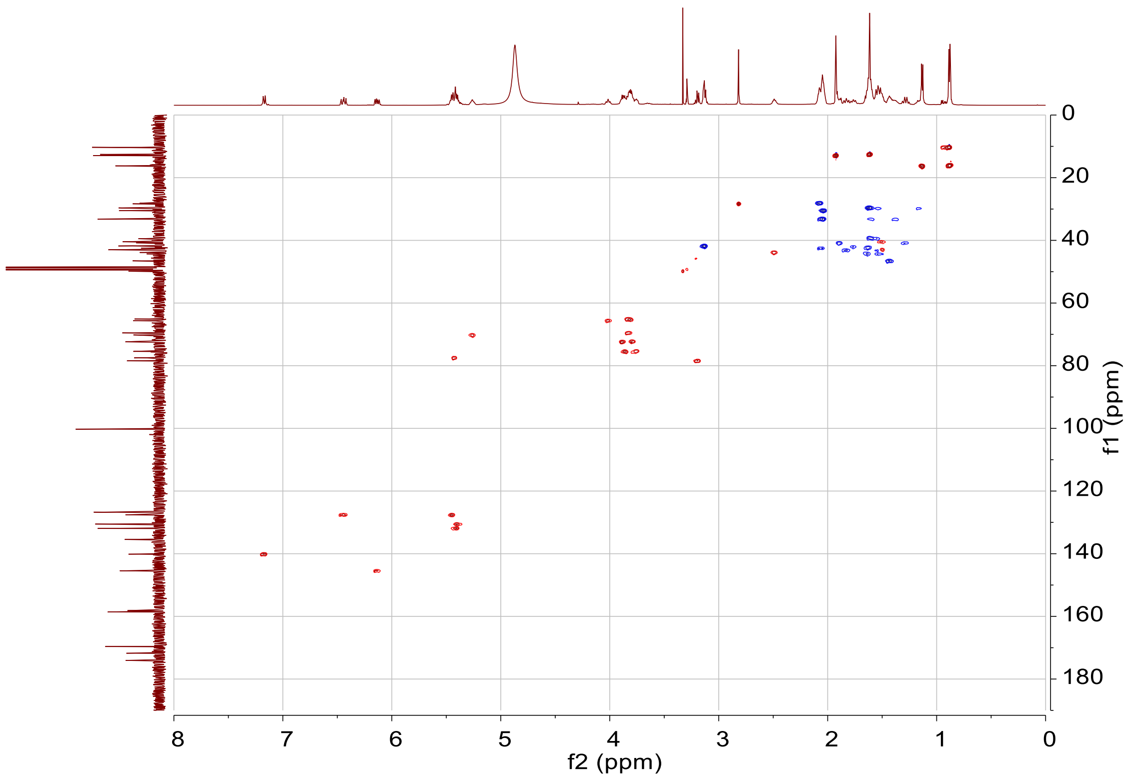

Supplementary Fig. 24. HSQC spectrum (600 MHz, CD_3OD) of compound **3**.

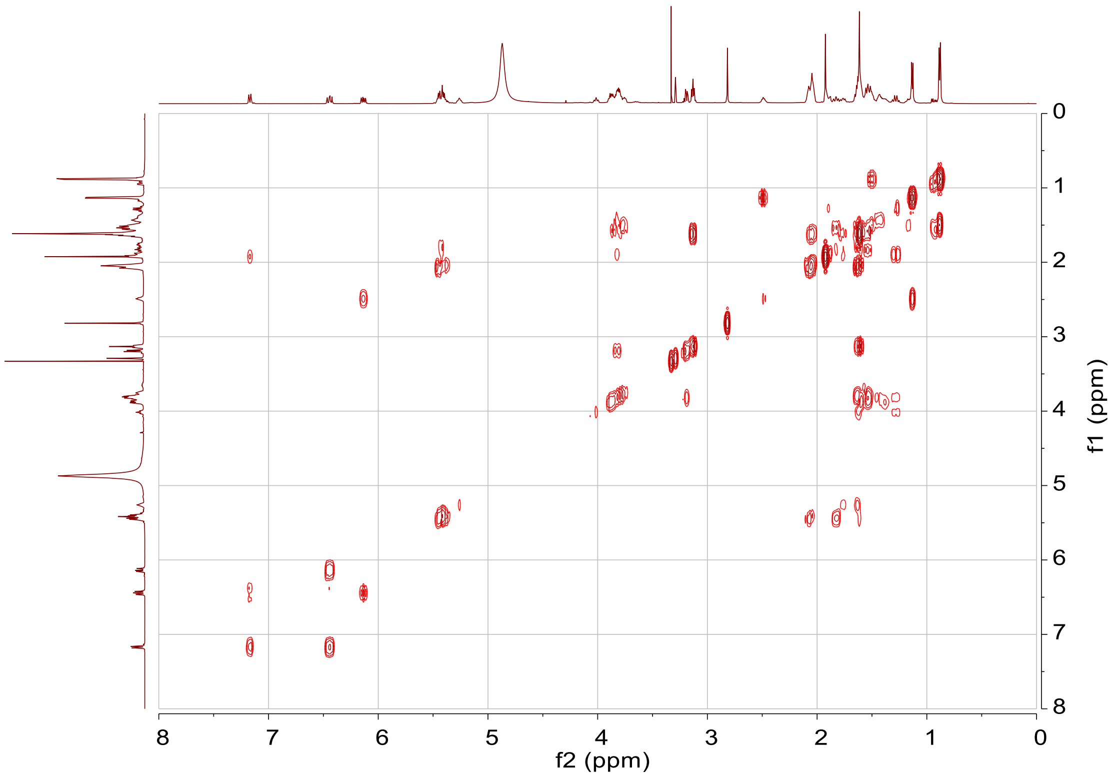

Supplementary Fig. 25. ^1H-^1H COSY spectrum (600 MHz, CD_3OD) of compound **3**.

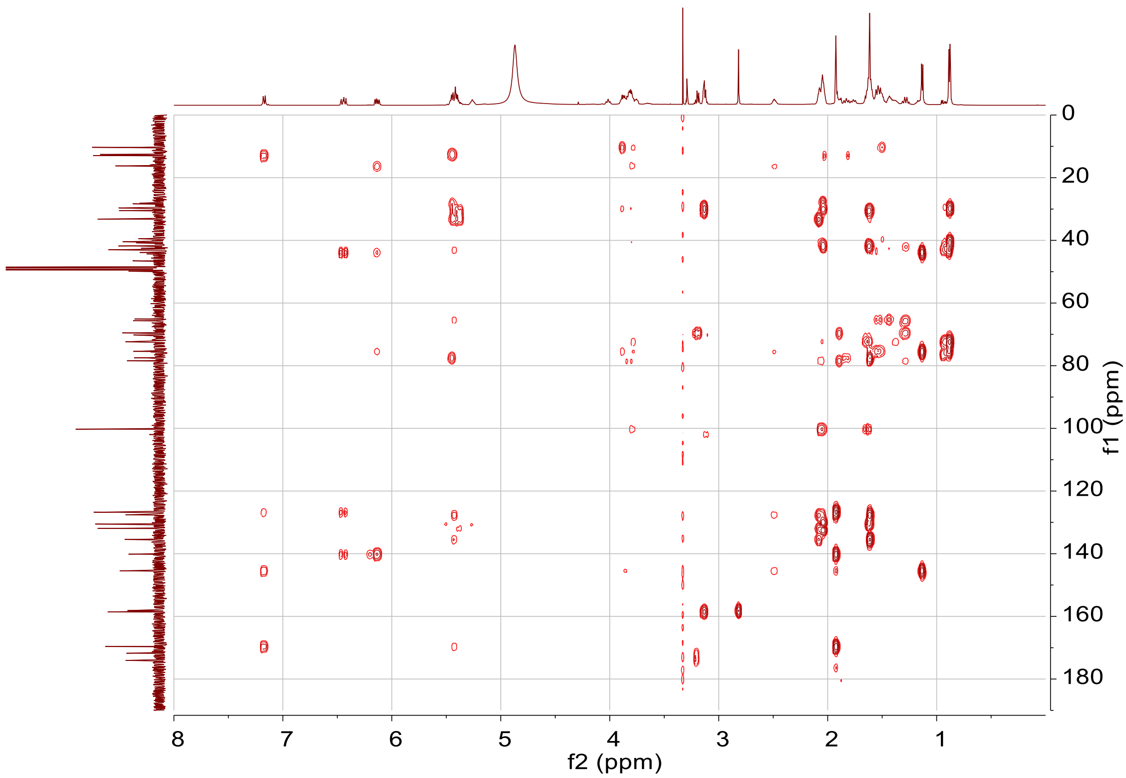

Supplementary Fig. 26. HMBC spectrum (600 MHz, CD_3OD) of compound **3**.

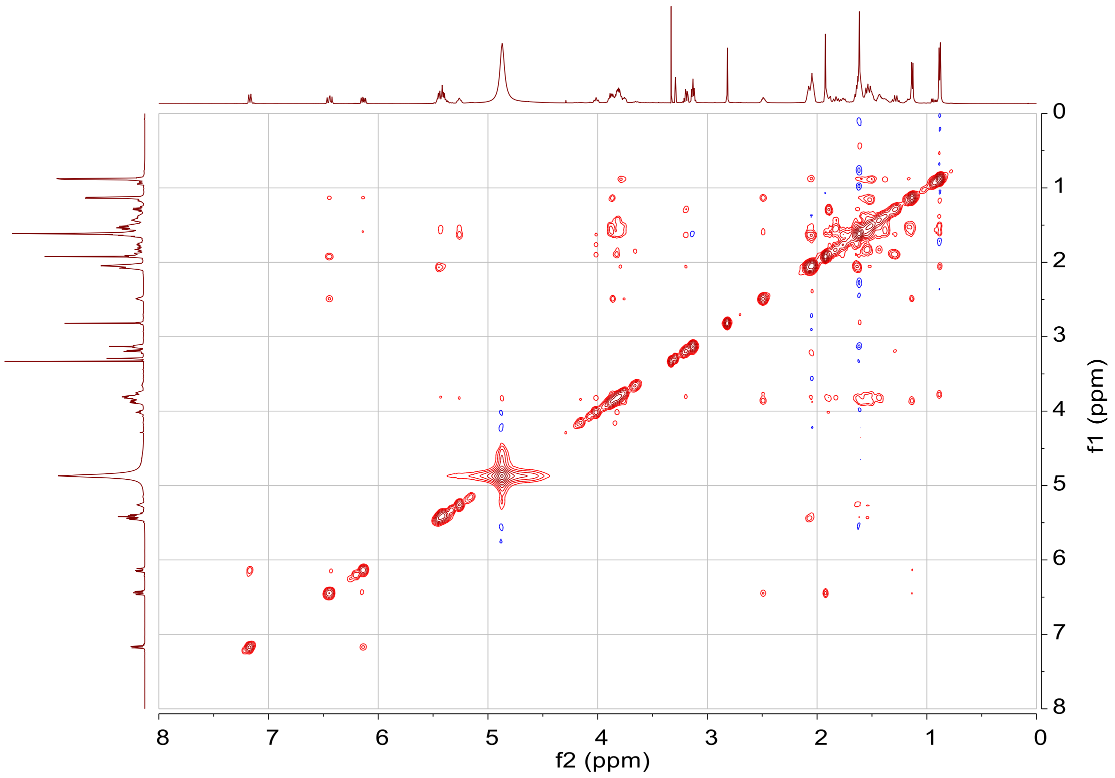

Supplementary Fig. 27. NOESY spectrum (600 MHz, CD_3OD) of compound **3**.

Supplementary Note 4. NMR spectra of compound **4**.

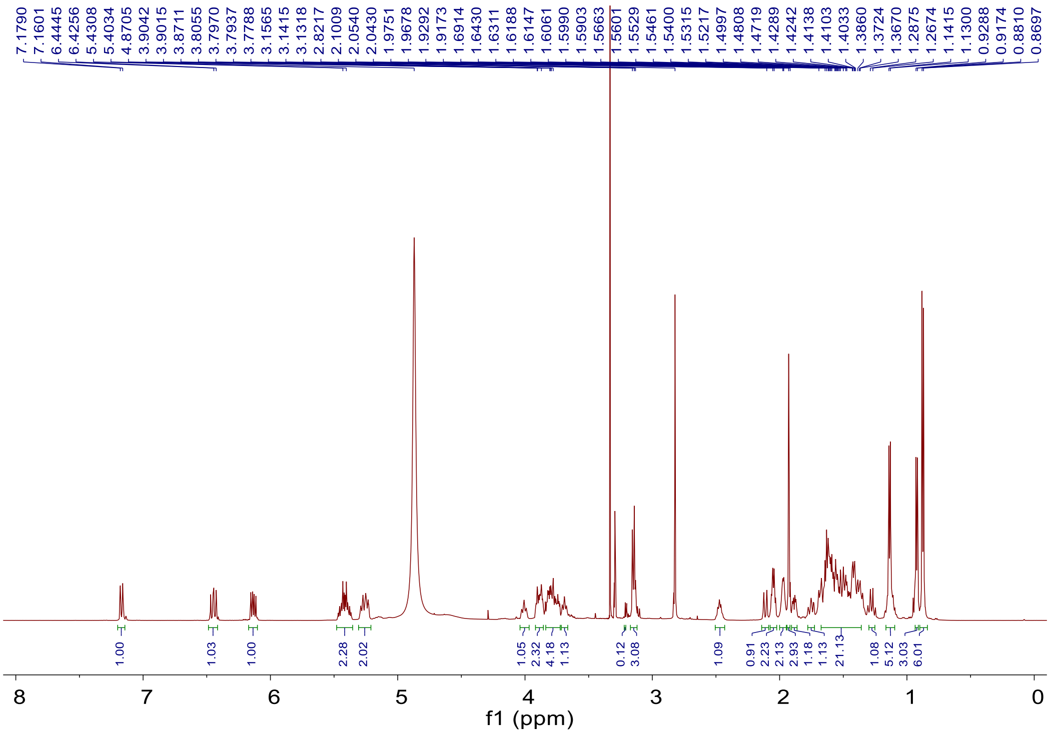

Supplementary Fig. 28. ^{1}H NMR spectrum (600 MHz, CD_3OD) of compound **4**.

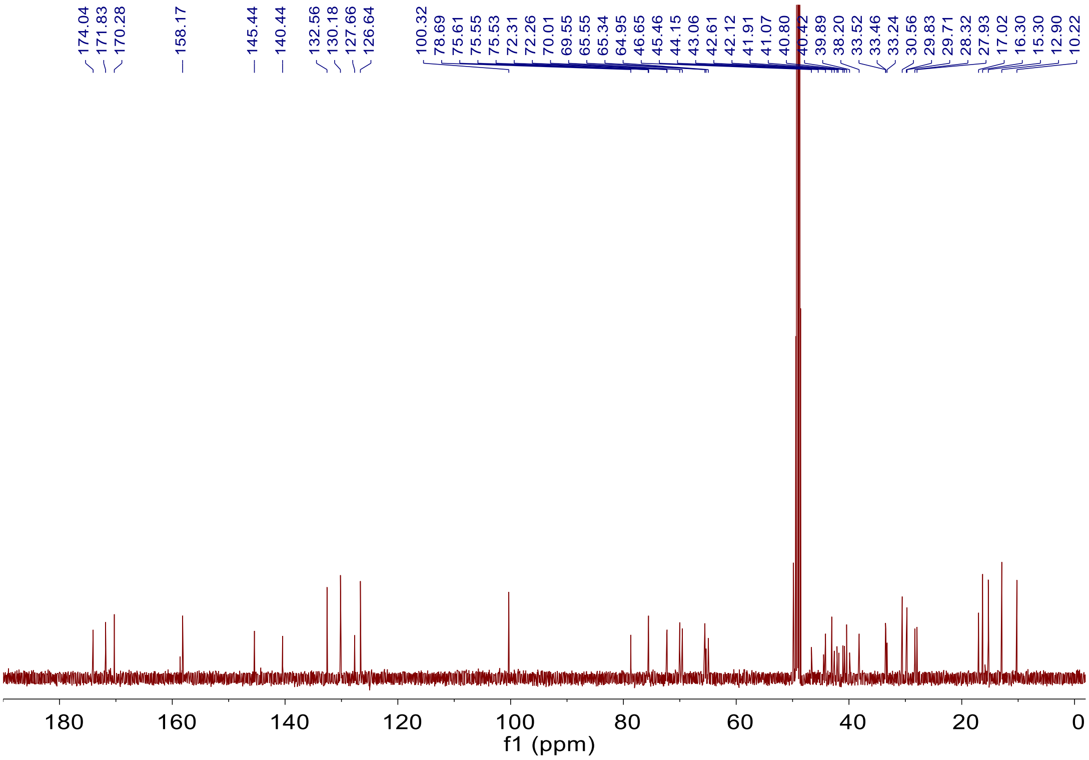

Supplementary Fig. 29. ^{13}C NMR spectrum (150 MHz, CD_3OD) of compound **4**.

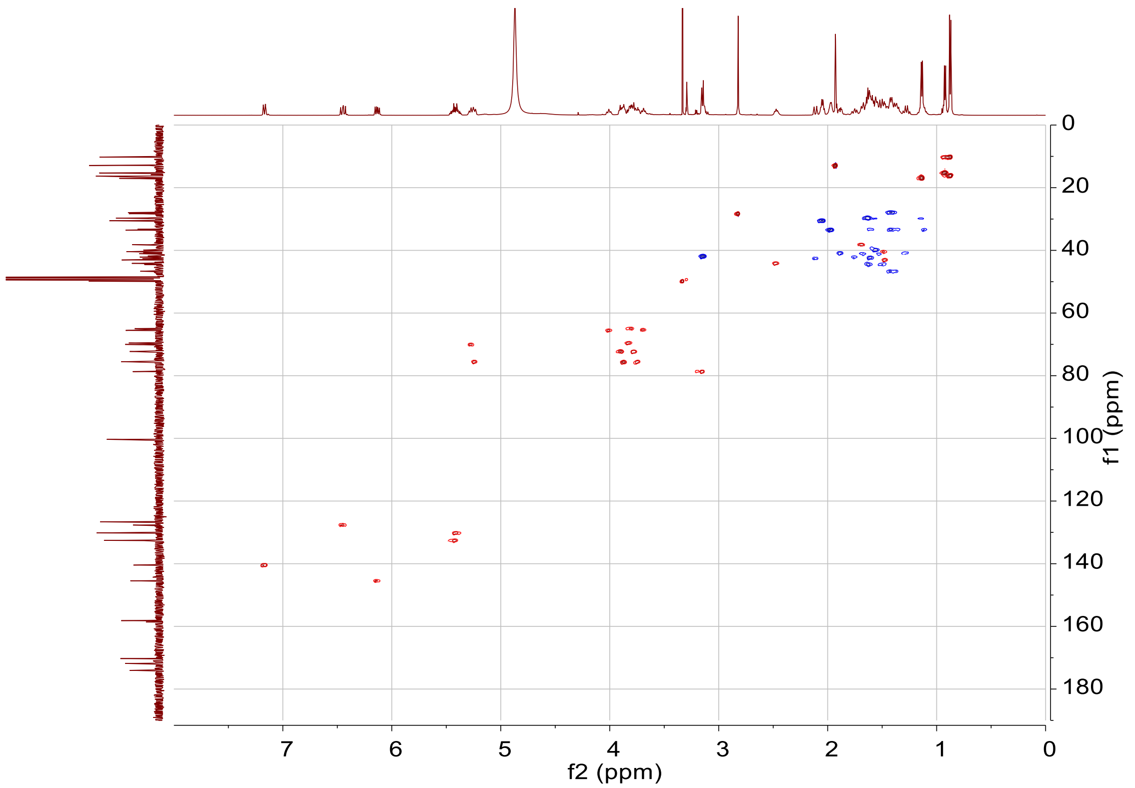

Supplementary Fig. 30. HSQC spectrum (600 MHz, CD_3OD) of compound **4**.

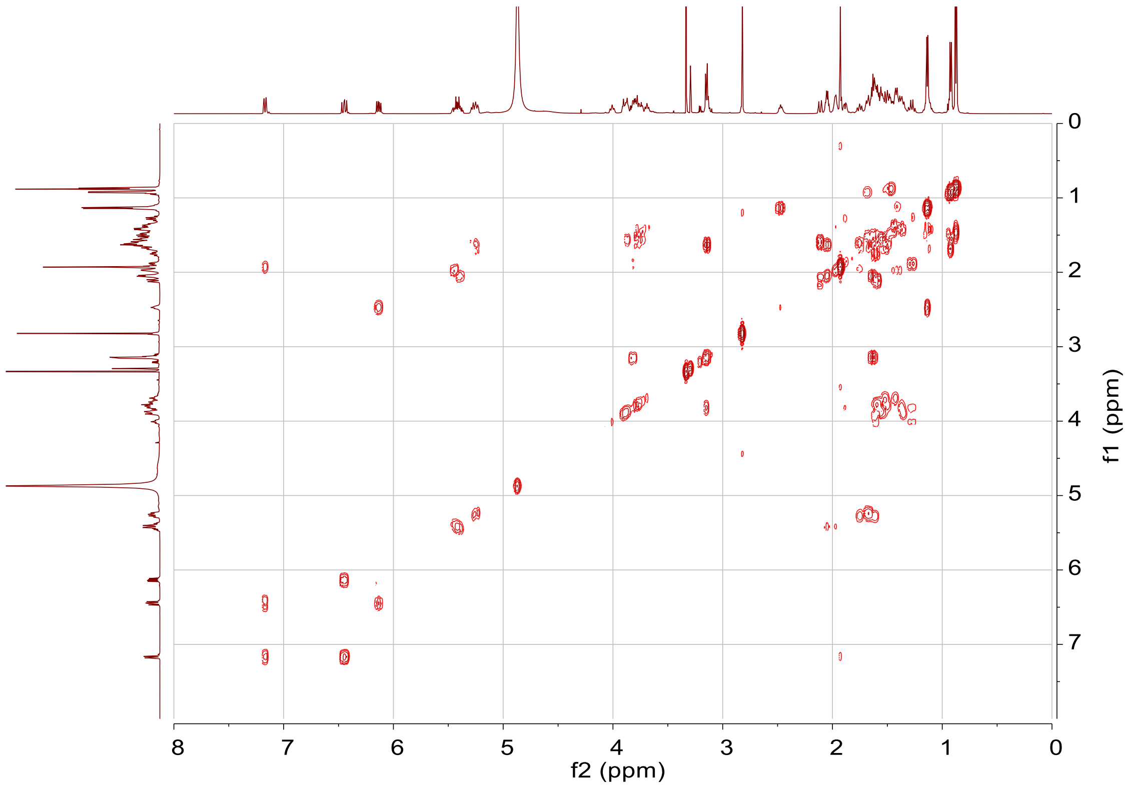

Supplementary Fig. 31. ^{1}H-^{1}H COSY spectrum (600 MHz, CD_3OD) of compound **4**.

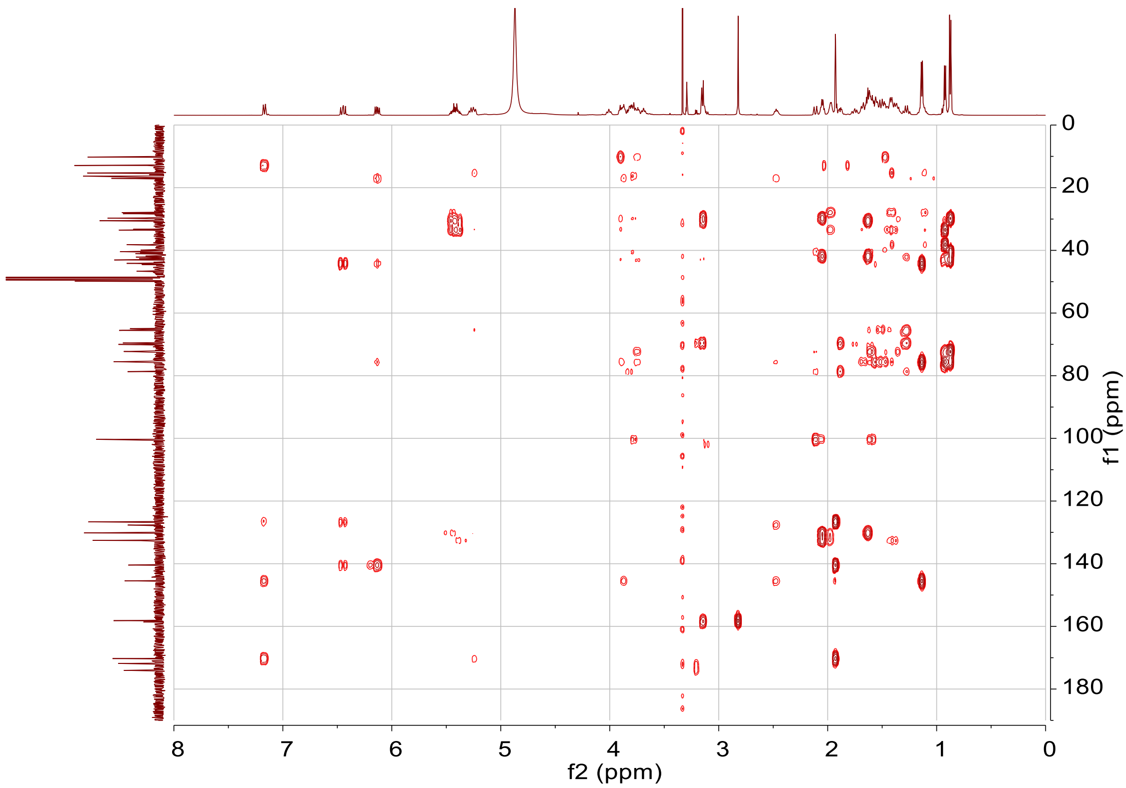

Supplementary Fig. 32. HMBC spectrum (600 MHz, CD_3OD) of compound **4**.

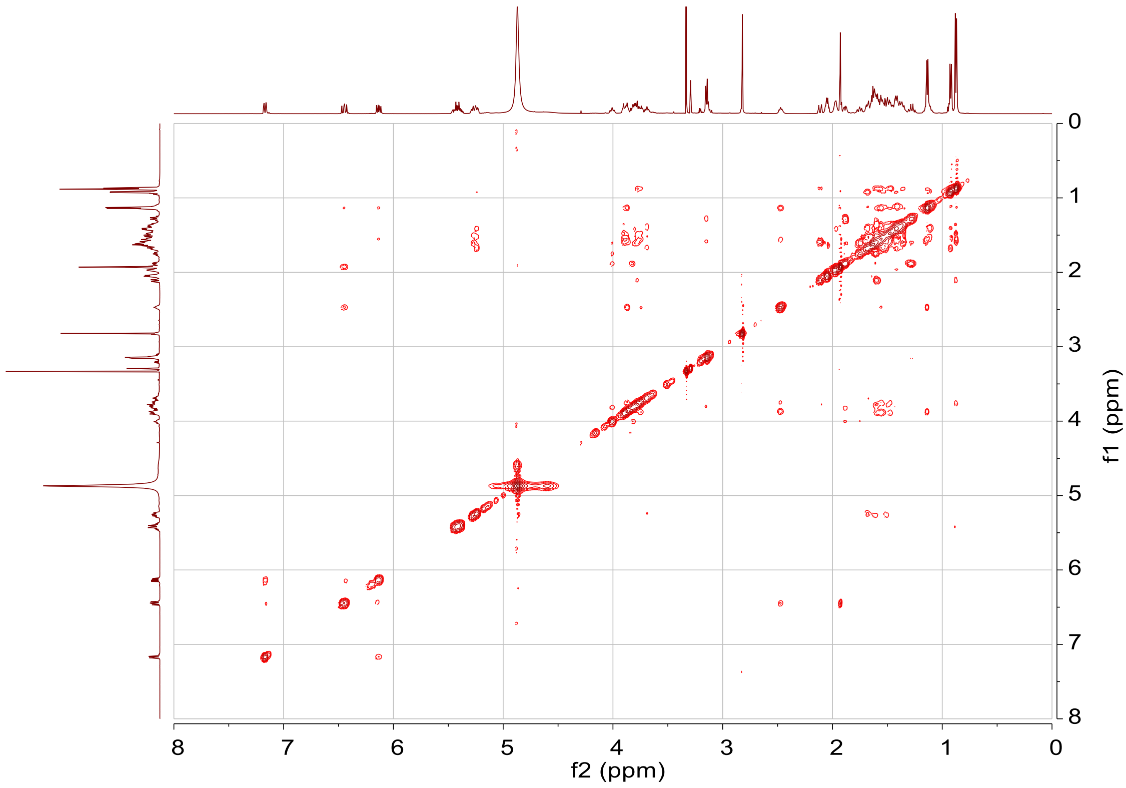

Supplementary Fig. 33. NOESY spectrum (600 MHz, CD_3OD) of compound **4**.

Supplementary References

1. MacNeil, D. J. et al. Analysis of *Streptomyces avermitilis* genes required for avermectin biosynthesis utilizing a novel integration vector. *Gene* **111**, 61–68 (1992).

2. Meng, W. & Jin, W. Z. Structure determination of new antifungal antibiotics, polaramycins A and B. *Acta Pharm. Sin.* **32**, 352–356 (1997).

3. Yuan, G. Lin, H. Wang, C. Hong, K. Liu, Y & Li, J. ^{1}H and ^{13}C assignments of two new macrocyclic lactones isolated from *Streptomyces* sp. 211726 and revised assignments of azalomycins F3a, F4a and F5a. *Magn. Reson. Chem.* **49**, 30–37 (2011).

4. Yuan, G. Hong, K. Lin, H. She, Z & Li, J. New azalomycin F analogs from mangrove *Streptomyces* sp. 211726 with activity against microbes and cancer cells. *Mar. Drugs* **11**, 817–829 (2013).

5. Sun, Y., He, X., Liang, J., Zhou, X. & Deng, Z. Analysis of functions in plasmid pHZ1358 influencing its genetic and structural stability in *Streptomyces lividans* 1326. *Appl. Microbiol*. *Biotechnol*. **82**, 303–310 (2009).

6. Jumper, J. *et al*. Highly accurate protein structure prediction with AlphaFold. *Nature* **596**, 583–589 (2021).
