# Supplementary material for: Insights into azalomycin F assembly-line contribute to evolution-guided polyketide synthase engineering and identification of intermodular recognition
Source: Nat Commun. 2023 Feb 4;14:612. doi: 10.1038/s41467-023-36213-9 (PMC9899208; doi:10.1038/s41467-023-36213-9)
Supplement: Supplementary file 3 — Reporting Summary [file 41467_2023_36213_MOESM3_ESM.pdf]

nature portfolio

Corresponding author(s): Yuhui Sun

Last updated by author(s): Jan 11, 2023

Reporting Summary

Nature Portfolio wishes to improve the reproducibility of the work that we publish. This form provides structure for consistency and transparency in reporting. For further information on Nature Portfolio policies, see our Editorial Policies and the Editorial Policy Checklist.

Statistics

For all statistical analyses, confirm that the following items are present in the figure legend, table legend, main text, or Methods section.

n/a	Confirmed	
☐	☒	The exact sample size (*n*) for each experimental group/condition, given as a discrete number and unit of measurement
☐	☒	A statement on whether measurements were taken from distinct samples or whether the same sample was measured repeatedly
☒	☐	The statistical test(s) used AND whether they are one- or two-sided<br>*Only common tests should be described solely by name; describe more complex techniques in the Methods section.*
☒	☐	A description of all covariates tested
☒	☐	A description of any assumptions or corrections, such as tests of normality and adjustment for multiple comparisons
☒	☐	A full description of the statistical parameters including central tendency (e.g. means) or other basic estimates (e.g. regression coefficient) AND variation (e.g. standard deviation) or associated estimates of uncertainty (e.g. confidence intervals)
☒	☐	For null hypothesis testing, the test statistic (e.g. *F*, *t*, *r*) with confidence intervals, effect sizes, degrees of freedom and *P* value noted<br>*Give P values as exact values whenever suitable.*
☒	☐	For Bayesian analysis, information on the choice of priors and Markov chain Monte Carlo settings
☒	☐	For hierarchical and complex designs, identification of the appropriate level for tests and full reporting of outcomes
☒	☐	Estimates of effect sizes (e.g. Cohen's *d*, Pearson's *r*), indicating how they were calculated

Our web collection on statistics for biologists contains articles on many of the points above.

Software and code

Policy information about availability of computer code

Data collection	No software was used for data collection
Data analysis	MEGA-CC version 11.0.11 for gene evolution analysis<br>MestReNova version 9.0.1-13254 for compounds structure characterization<br>ChemBioDraw version 14.0.0.117 for drawing compounds structure<br>BioEdit version 7.2.6 for sequence alignment

For manuscripts utilizing custom algorithms or software that are central to the research but not yet described in published literature, software must be made available to editors and reviewers. We strongly encourage code deposition in a community repository (e.g. GitHub). See the Nature Portfolio guidelines for submitting code & software for further information.

Data

Policy information about availability of data

All manuscripts must include a data availability statement. This statement should provide the following information, where applicable:

- Accession codes, unique identifiers, or web links for publicly available datasets
- A description of any restrictions on data availability
- For clinical datasets or third party data, please ensure that the statement adheres to our policy

The data generated in this study are provided in the Supplementary Information and Source Data files provided with this paper. Further data, plasmids and primers

are also available from the corresponding author upon request.

Human research participants

Policy information about studies involving human research participants and Sex and Gender in Research.

Reporting on sex and gender	Not applicable
Population characteristics	Not applicable
Recruitment	Not applicable
Ethics oversight	Not applicable

Note that full information on the approval of the study protocol must also be provided in the manuscript.

Field-specific reporting

Please select the one below that is the best fit for your research. If you are not sure, read the appropriate sections before making your selection.

☒ Life sciences ☐ Behavioural & social sciences ☐ Ecological, evolutionary & environmental sciences

For a reference copy of the document with all sections, see nature.com/documents/nr-reporting-summary-flat.pdf

Life sciences study design

All studies must disclose on these points even when the disclosure is negative.

Sample size	No statistical methods were used in this study
Data exclusions	No data were excluded
Replication	All engineered mutants were repeatedly confirmed during the process of mutants screening and fermentation using PCR and sequencing. All strains used in this study were fermented in triplicate, and the fermentation events independently repeated several times with the similar results.
Randomization	Specific controls were rationally designed in each group of experiment to draw a conclusion and dissect the mechanism of cross-module enoylreduction. For example, there are only two ER domains (ER1/2 and ER15) in the AZL PKS assembly-line. It is rational to design the specific replacement experiment using ER15 to replace ER1/2 to investigate the correlation between the unusual behaviors and characteristics of ER1/2. Moreover, the fermentation result of ΔazlB showed that some divergence between modules 3 and 6 must be the reason for ER1/2 recruitment, which is not present in module 6. It is most reasonable to replace each domain of module 6 with the corresponding domain of module 3 in the ΔazlB mutant rather than select domains randomly from other modules to characterize the difference between module 3 and 6. Therefore, randomization is not applicable in this study.
Blinding	Based on bioinformatic results, we designed a series of genetic experiments (such as gene or domain deletion and replacement) to investigate the function of domains and modules in AZL PKS assembly-line, so blinding is not applicable in this study.

Reporting for specific materials, systems and methods

We require information from authors about some types of materials, experimental systems and methods used in many studies. Here, indicate whether each material, system or method listed is relevant to your study. If you are not sure if a list item applies to your research, read the appropriate section before selecting a response.

Materials & experimental systems

n/a	Involved in the study	
☒	☐	Antibodies
☒	☐	Eukaryotic cell lines
☒	☐	Palaeontology and archaeology
☒	☐	Animals and other organisms
☒	☐	Clinical data
☒	☐	Dual use research of concern

Methods

n/a	Involved in the study	
☒	☐	ChIP-seq
☒	☐	Flow cytometry
☒	☐	MRI-based neuroimaging
